# Quality Assessment in Frozen Seafood: Advances in Sensing Technologies and Artificial Intelligence

**DOI:** 10.3390/foods15162799

**Published:** 2026-08-10

**Authors:** Mubeen Tageldin Omer Mohamed, Xorlali Nunekpeku, Nama Yaa Akyea Prempeh, Wenjing Jiang, Huanhuan Li

**Affiliations:** Food Science and Engineering College, Jiangsu University, Zhenjiang 212013, China; mubeen@stmail.ujs.edu.cn (M.T.O.M.); 2222418045@stmail.ujs.edu.cn (W.J.)

**Keywords:** frozen seafood, non-destructive sensing, artificial intelligence, cold-chain monitoring, spectroscopic analysis, data fusion

## Abstract

Frozen seafood plays an important role in the global food supply, but maintaining its quality during frozen storage and cold-chain distribution remains a significant challenge. Although freezing effectively slows microbial growth and enzymatic activity, it cannot completely prevent quality deterioration. During frozen storage, seafood undergoes a series of interconnected physicochemical changes, including ice crystal growth, protein denaturation and oxidation, lipid oxidation, water redistribution, and texture deterioration. These changes gradually reduce sensory quality, nutritional value, and overall commercial acceptability. Conventional quality assessment methods, including destructive laboratory analyses and sensory evaluation, are still widely used. However, they are often labor-intensive, time-consuming, and unsuitable for rapid or real-time monitoring in modern cold-chain systems. As a result, increasing attention has been given to non-destructive sensing technologies that can evaluate seafood quality quickly and objectively. This review summarizes the major mechanisms responsible for quality deterioration in frozen seafood, together with recent advances in sensing technologies used to monitor these changes. The sensing approaches discussed include near-infrared (NIR) and Raman spectroscopy, hyperspectral and fluorescence imaging, low-field nuclear magnetic resonance (LF-NMR), electronic nose (E-nose), electronic tongue (E-tongue), colorimetric sensor arrays (CSAs), and biosensors. This review also discusses the growing role of artificial intelligence in frozen seafood quality assessment, including chemometrics, machine learning, deep learning, and multi-sensor data fusion. Particular attention is given to their applications in quality prediction, industrial implementation, and decision support. Finally, current challenges and future research needs are highlighted, with emphasis on the development of interpretable, transferable, and real-time monitoring systems that can support more reliable quality assurance throughout the frozen seafood supply chain.

## 1. Introduction

The global seafood industry plays a vital role in food security and human nutrition by supplying high-quality protein, long-chain omega-3 polyunsaturated fatty acids, essential vitamins, and trace minerals to billions of people worldwide [1,2,3,4,5]. With the continued expansion of aquaculture, increasing international trade, and rising consumer demand for safe and convenient foods, freezing has become the preferred preservation method for extending shelf life, reducing post-harvest losses, and enabling the global distribution of seafood products [1,6,7].

Although freezing effectively inhibits microbial growth and considerably slows enzymatic activity, it cannot completely prevent quality deterioration. During frozen storage and repeated freeze–thaw (F-T) cycles, seafood experiences a series of interconnected physical, chemical, and biochemical changes. These include ice crystal formation and recrystallization, protein denaturation and oxidation, lipid oxidation, water redistribution, increased drip loss, texture softening, and pigment degradation [7,8,9]. Many of these changes develop gradually and are difficult to detect by visual inspection. Conventional seafood quality evaluation mainly relies on destructive laboratory analyses, including total volatile basic nitrogen (TVB-N), thiobarbituric acid reactive substances (TBARS), K value, and aerobic plate count, together with sensory evaluation by trained panels [10,11,12]. While these techniques remain the benchmark for quality assessment, they require laboratory facilities, are labor-intensive, and are often too slow for routine monitoring.

In recent years, non-destructive sensing technologies have provided new opportunities for monitoring seafood quality more rapidly and objectively. Techniques such as near-infrared (NIR) spectroscopy, Raman spectroscopy, hyperspectral imaging (HSI), fluorescence spectroscopy, low-field nuclear magnetic resonance (LF-NMR), electronic nose (E-nose), electronic tongue (E-tongue), colorimetric sensor arrays (CSAs), and biosensors can detect changes in chemical composition, water distribution, tissue structure, oxidation, and volatile compounds without damaging the sample [13,14,15,16,17,18]. Chemometric techniques, machine learning, deep learning, and multi-sensor data fusion are now widely applied to extract useful information from sensing data and improve the prediction of quality attributes [19,20,21].

Despite these advances, research on frozen seafood remains relatively fragmented. Quality evaluation in frozen seafood is particularly challenging because deterioration is influenced not only by storage time but also by ice crystal formation, freeze concentration, repeated freeze–thaw cycles, and the complex interactions among multiple deterioration mechanisms. In addition, many sensing technologies have only been evaluated under controlled laboratory conditions, with limited validation under real industrial cold-chain environments.

**Figure 1:**
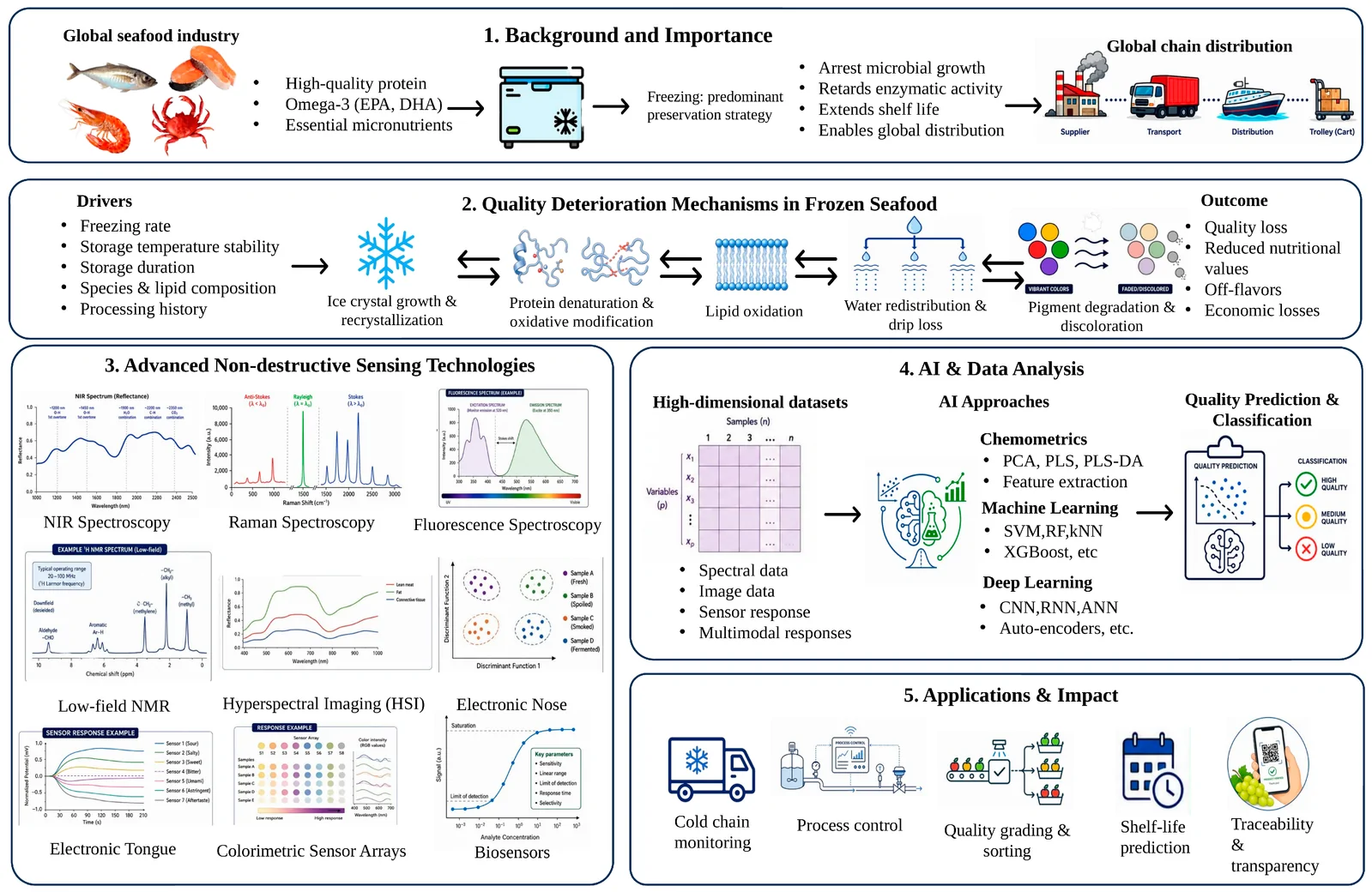
Conceptual framework for frozen seafood quality assessment using advanced sensing technologies and artificial intelligence. The figure summarizes the background and importance of frozen seafood preservation, major quality deterioration mechanisms, representative non-destructive sensing technologies, AI and data-analysis approaches, and practical applications for cold-chain monitoring, process control, quality grading, shelf-life prediction, and traceability. Arrows shows the flow and the connection between the different part. Bidirectional arrows show interactions between deterioration mechanisms.

This review aims to address these issues by providing a comprehensive overview of recent developments in frozen seafood quality monitoring. Specifically, it (i) examines the physicochemical and biochemical mechanisms responsible for quality deterioration, with emphasis on ice crystal damage, protein denaturation and oxidation, lipid oxidation, and water redistribution; (ii) reviews recent advances in non-destructive sensing technologies, including their principles, advantages, limitations, and potential for industrial application; (iii) discusses current AI-based approaches for quality prediction, including chemometrics, machine learning, deep learning, and multi-sensor data fusion; and (iv) highlights current challenges and future research priorities for developing practical, interpretable, and real-time quality monitoring systems. The overall framework of this review is illustrated in Figure 1.

## 2. Quality Deterioration Mechanisms in Frozen Seafood

Frozen storage is widely used to extend the shelf life of seafood by inhibiting microbial growth and slowing enzymatic and chemical reactions. However, freezing does not completely prevent quality deterioration. Instead, the freezing process itself, together with prolonged frozen storage, gradually introduces a series of physical, chemical, and biochemical changes that reduce product quality over time [22,23]. The extent of these changes depends on several interacting factors, including freezing rate, storage temperature and its stability, storage duration, species characteristics such as lipid content and muscle fiber structure, and processing conditions before freezing, including handling and packaging [7,24].

The major deterioration pathways include ice crystal formation and recrystallization, protein denaturation and oxidation, lipid oxidation, water migration and redistribution, drip loss, texture softening, and pigment degradation. These processes are closely interconnected rather than occurring independently, and their interactions often accelerate the overall deterioration of frozen seafood during storage [24,25].

Understanding how these quality changes develop is essential for effective quality control throughout frozen storage and distribution. This section reviews the main quality attributes of frozen seafood together with the key deterioration mechanisms, providing a scientific basis for selecting appropriate sensing indicators and developing reliable non-destructive monitoring technologies.

### 2.1. Key Quality Attributes

Frozen seafood quality is assessed using a combination of physical, chemical, microbiological, and sensory attributes. Physical characteristics, including texture, water-holding capacity (WHC), drip loss, and muscle microstructure, are strongly influenced by ice crystal formation and the functional properties of muscle proteins [24,26,27]. Among the chemical indicators, protein and lipid oxidation are particularly important because they are closely associated with quality deterioration during frozen storage.

While microbial activity is largely suppressed under frozen conditions, spoilage microorganisms may become active again after thawing or when products are exposed to temperature abuse during distribution. Sensory properties, including color, odor, texture, and overall acceptability, reflect the combined effects of these physical and biochemical changes and remain the primary factors influencing consumer acceptance and commercial value. Recent kinetic modeling studies further show that texture softening and color fading follow predictable changes during storage and are closely related to storage time and pre-treatment conditions, highlighting their value as quantitative quality indicators [28]. Texture and color are therefore the most practically tractable targets for non-destructive sensing approaches.

In summary, frozen seafood quality is determined by the interaction of physical, chemical, and sensory attributes. Among these, texture and color are particularly sensitive to storage-related deterioration and therefore serve as important targets for quality monitoring using non-destructive sensing technologies.

### 2.2. Protein Oxidation and Denaturation

**Figure 2:**
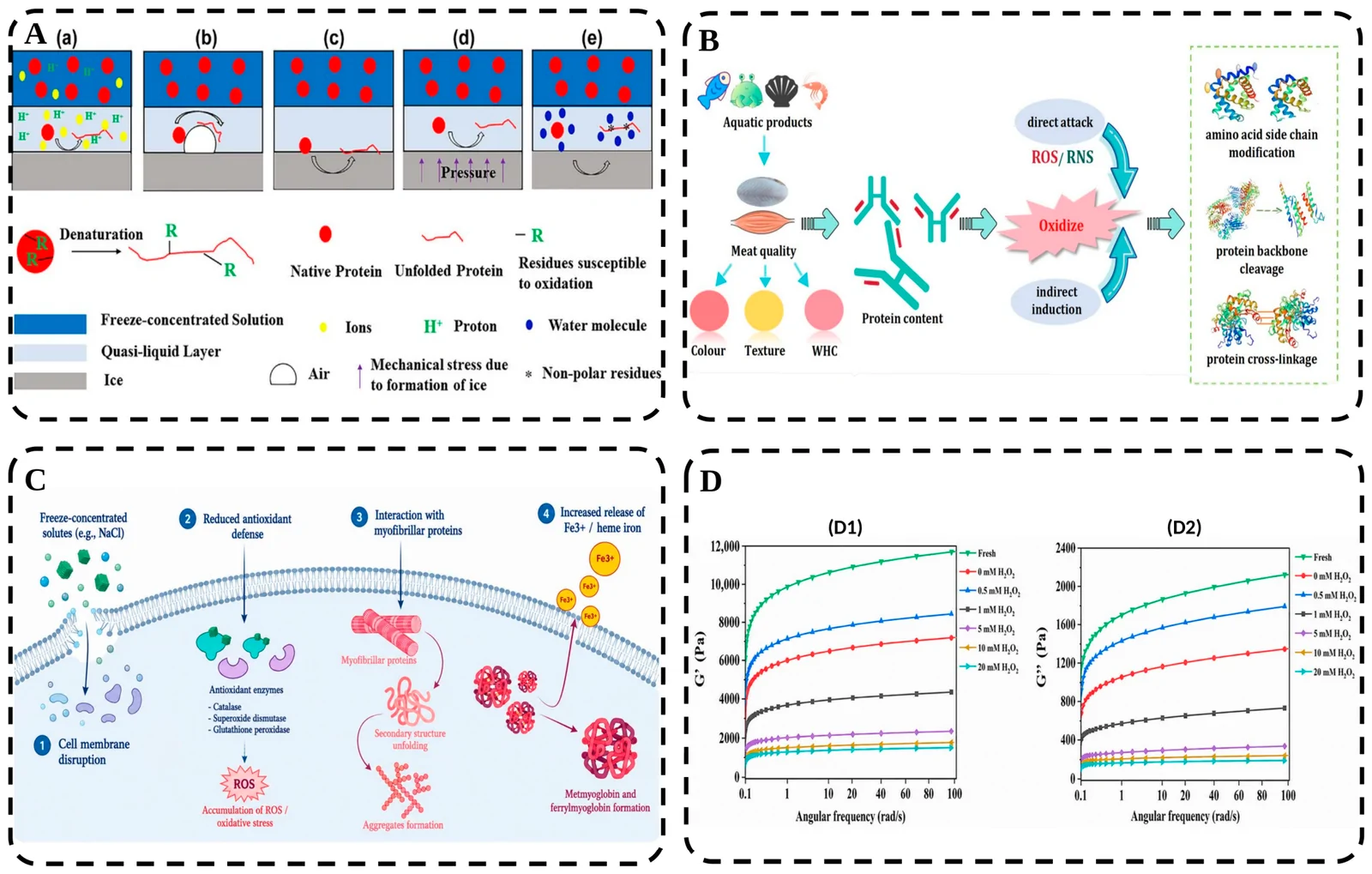
Mechanisms of protein denaturation and oxidation in frozen seafood. (**A**) possible mechanisms of ice induced protein denaturation: (a) Change of pH and ionic strength. (b) Accumulation of air bubbles at the ice interface. (c) Adsorption at the ice interface. (d) Pressure due to ice growth. (e) Enhance cold denaturation. (**B**) Reactive oxygen and nitrogen species induce protein oxidation through amino acid modification, peptide backbone cleavage, and protein cross-linking. (**C**) Ice crystal-induced membrane disruption enhances oxidative stress, leading to antioxidant depletion, protein aggregation, and the release of pro-oxidative iron species. (**D**) Oxidation-dependent changes in myofibrillar protein gelation: (D1) storage modulus (G′) and (D2) loss modulus (G″) of myofibrillar protein gel. Adapted from [25,47,48,49].

Protein denaturation is one of the primary biochemical processes responsible for quality deterioration in frozen seafood. It results from the combined effects of physical damage caused by freezing and oxidative reactions that continue throughout frozen storage and subsequent thawing [6,29,30]. Ice crystal formation and recrystallization disrupt the native three-dimensional structure of muscle proteins, particularly the myofibrillar proteins myosin, actin, and tropomyosin. As these proteins partially unfold, previously buried hydrophobic regions become exposed, increasing the likelihood of intermolecular aggregation and cross-linking [31]. These structural changes reduce important functional properties, especially water-holding capacity and gel-forming ability, which ultimately lead to greater drip loss, firmer texture, and reduced tenderness in frozen seafood [6,32,33]. The extent of protein damage depends largely on freezing conditions. Rapid freezing produces numerous small ice crystals that are distributed more uniformly within the muscle, minimizing structural disruption. In contrast, slow freezing promotes the formation of larger extracellular ice crystals that rupture cell membranes and damage muscle fibers [24]. During prolonged storage, fluctuations in temperature accelerate ice recrystallization through Ostwald ripening, where smaller ice crystals gradually disappear while larger ones continue to grow. As a result, mechanical damage to muscle tissue becomes more severe over time [6,25]. Figure 2 summarizes the sequence of events linking freezing-induced structural damage with protein oxidation. As illustrated in Figure 2A, freeze concentration and ice crystal formation expose hydrophobic regions and amino acid residues that are particularly susceptible to oxidation. These structural changes increase the vulnerability of proteins to reactive oxygen species, which subsequently promote amino acid side-chain oxidation, peptide backbone cleavage, and protein cross-linking (Figure 2B). At the same time, membrane disruption caused by ice crystals releases pro-oxidative iron species while reducing endogenous antioxidant protection, further accelerating protein aggregation and oxidative deterioration (Figure 2C). Figure 2D presents rheological evidence that different oxidation levels alter myofibrillar protein gelation, as reflected by changes in the storage modulus (G′), loss modulus (G″), and overall gel-forming ability.

As oxidation progresses, the structural and functional properties of muscle proteins continue to decline [34,35]. Typical indicators include carbonyl accumulation, sulfhydryl loss, increased surface hydrophobicity, altered water distribution, and reduced water-holding capacity, all of which contribute to deterioration in texture and overall functionality during frozen storage [25,36,37,38,39]. These structural changes manifest as increased hardness, decreased elasticity, and loss of the characteristic texture properties valued in fresh seafood products [25].

These molecular changes are reflected in poorer eating quality, with frozen seafood becoming harder, less elastic, and less capable of retaining moisture after thawing. Oxidative damage is largely driven by reactive oxygen species, which preferentially attack amino acid residues such as methionine, histidine, lysine, and cysteine. These reactions generate carbonyl compounds, promote disulfide bond formation, consume sulfhydryl groups, and encourage protein aggregation through covalent cross-linking [38,40,41,42,43]. Protein solubility decreases, enzyme activity is impaired, and important quality attributes such as tenderness, juiciness, and water-holding capacity continue to decline [29]. The rate of oxidation is further influenced by factors including residual oxygen, storage temperature, and secondary products generated during lipid oxidation [44]. Oxidation of myoglobin is another important consequence of frozen storage, particularly in red and dark-fleshed fish species. During storage, oxymyoglobin containing ferrous iron (Fe^2+^) is gradually converted to ferric metmyoglobin (Fe^3+^), causing the characteristic bright red color to fade into undesirable brown or gray tones that reduce consumer acceptance [45]. This process is accelerated by reactive aldehydes such as malondialdehyde (MDA), together with free iron released during lipid oxidation, highlighting the close relationship between protein and lipid oxidative pathways [46].

Overall, protein denaturation and oxidation occur simultaneously throughout frozen storage and together contribute to reduced water-holding capacity, texture deterioration, and color instability. Changes such as carbonyl accumulation, myoglobin oxidation, increased surface hydrophobicity, and protein aggregation therefore provide valuable indicators of frozen storage damage and serve as important targets for non-destructive quality assessment. Having an understanding of these mechanisms also provides a foundation for developing spectroscopic, imaging, and electrochemical sensing techniques capable of monitoring protein oxidation in real time.

### 2.3. Lipid Oxidation

Lipid oxidation is one of the major causes of quality deterioration in frozen seafood, particularly in fatty species rich in polyunsaturated fatty acids (PUFAs), such as eicosapentaenoic acid (EPA) and docosahexaenoic acid (DHA). Although freezing slows oxidative reactions, it cannot completely prevent them, allowing lipid oxidation to continue gradually throughout frozen storage [50,51]. During this process, primary oxidation products (lipid hydroperoxides) are formed and subsequently decompose into secondary compounds, including malondialdehyde (MDA), 4-hydroxynonenal (4-HNE), aldehydes, and ketones. These compounds contribute to rancid odors, off-flavors, nutritional losses, and reduced sensory quality [50,52].

Lipid oxidation is closely linked with other deterioration mechanisms. Reactive oxidation products, particularly aldehydes and hydroperoxides, readily interact with muscle proteins, promoting carbonyl formation, structural modification, and protein cross-linking, which further weakens muscle integrity [48].

**Figure 3:**
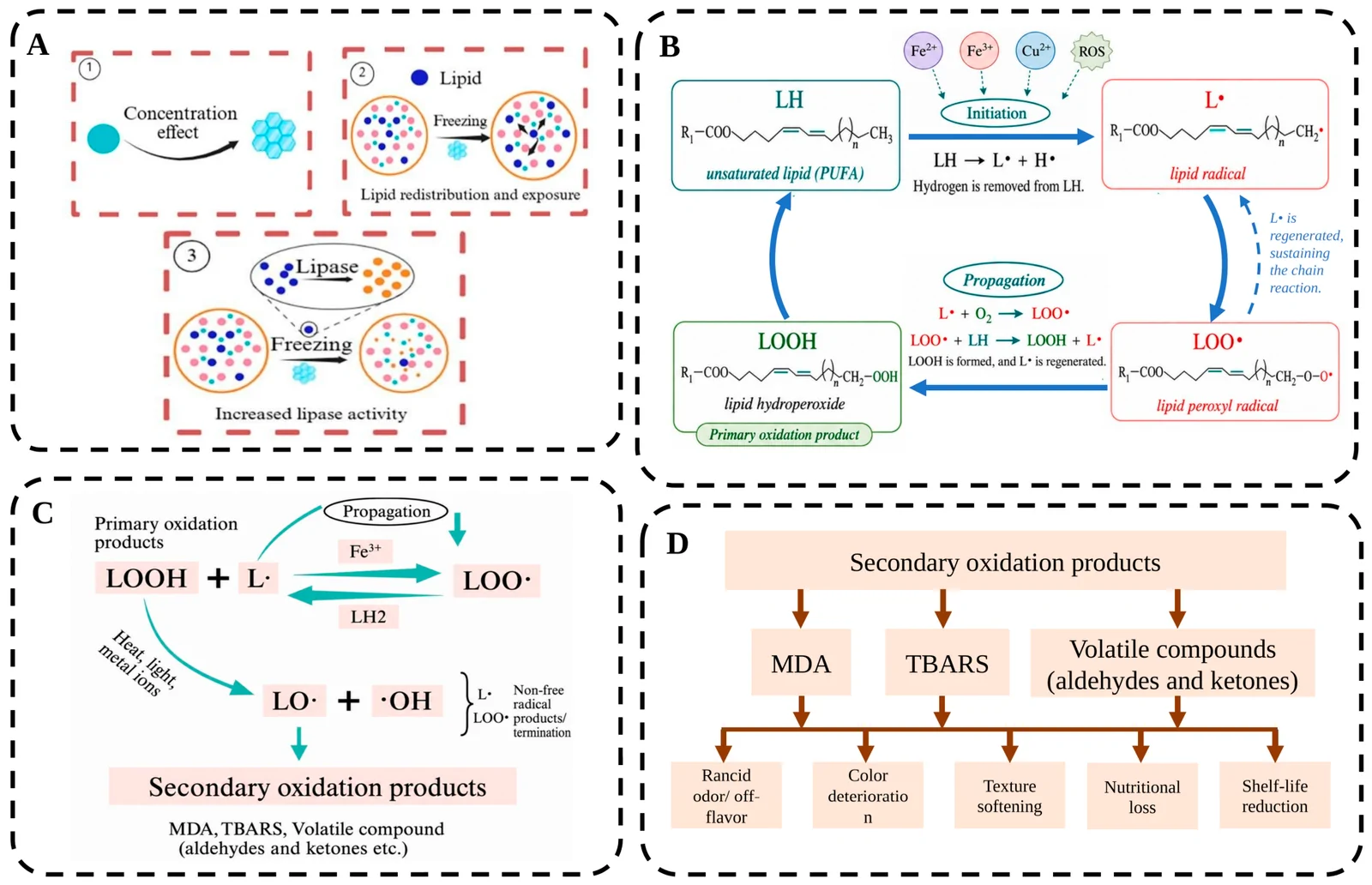
Mechanism of lipid oxidation in frozen seafood. (**A**) Freezing promotes lipid oxidation through freeze concentration, lipid redistribution, increased lipid exposure, and lipase activity. (**B**) Free-radical reactions generate lipid radicals, lipid peroxyl radicals, and lipid hydroperoxides as primary oxidation products. (**C**) Lipid hydroperoxides decompose into secondary oxidation products under the influence of heat, light, metal ions, and radical reactions. (**D**) Secondary oxidation products contribute to rancid odor, off-flavor development, discoloration, texture deterioration, nutritional loss, and reduced shelf life. Arrows indicate the direction of the processes and interactions illustrated. Adapted from [58].

Common quality indicators such as TBARS, peroxide value, and carbonyl content increase progressively during frozen storage and are widely used to evaluate oxidative deterioration [53,54,55,56]. Their sensitivity to storage temperature, packaging atmosphere, and lipid composition also makes them valuable targets for non-destructive sensing technologies. In addition, antioxidant treatments have been shown to reduce oxidation by lowering TBARS and protein carbonyl levels, preserving sulfhydryl groups, and improving water retention and texture stability [57]. Figure 3 illustrates the main pathway of lipid oxidation in frozen seafood. As shown in Figure 3A, freezing promotes lipid oxidation through freeze concentration, lipid redistribution, greater lipid exposure, and increased lipase activity. These changes facilitate free-radical reactions that generate lipid radicals, lipid peroxyl radicals, and lipid hydroperoxides as primary oxidation products (Figure 3B). The unstable hydroperoxides subsequently decompose into secondary products, including MDA, aldehydes, ketones, and other volatile compounds (Figure 3C). These compounds are directly associated with rancid odor, off-flavor development, discoloration, texture deterioration, nutritional loss, and reduced shelf life (Figure 3D). Lipid oxidation plays a central role in the deterioration of frozen seafood. Because it is closely associated with protein oxidation and sensory quality loss, lipid-derived oxidation products remain important indicators for quality assessment and represent key targets for non-destructive monitoring during frozen storage.

### 2.4. Ice Crystal Formation and Mechanical Damage

**Figure 4:**
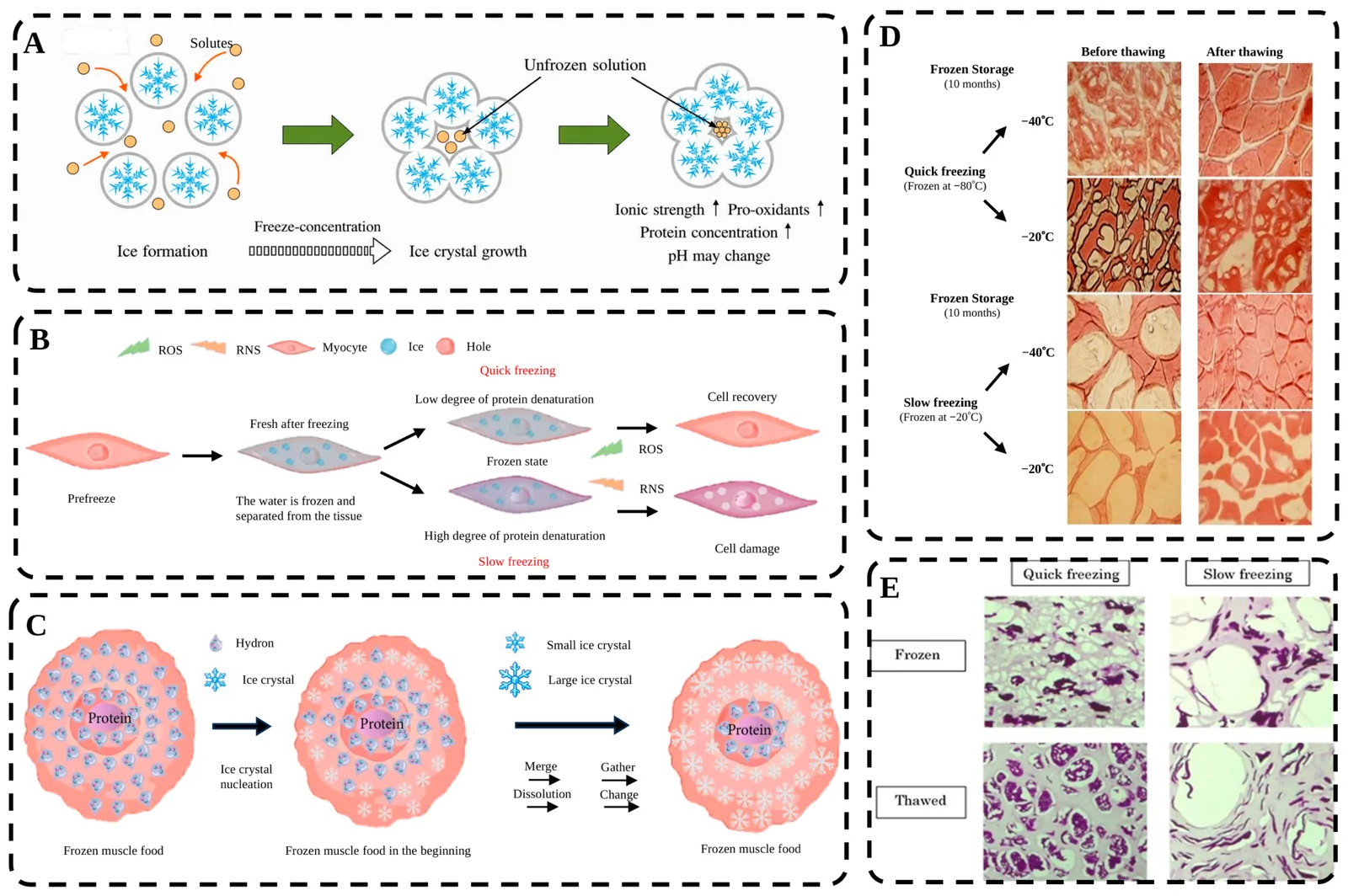
Ice crystal formation and microstructural damage in frozen seafood muscle. (**A**) Ice formation concentrates solutes in the unfrozen phase, increasing ionic strength, protein concentration, pro-oxidant levels, and local pH changes. (**B**) Rapid freezing produces smaller ice crystals with less protein damage, whereas slow freezing forms larger crystals that increase cell disruption and protein denaturation. (**C**) Ice crystal nucleation, growth, and recrystallization progressively damage muscle structure during frozen storage. (**D**,**E**) Microscopic images showing the effects of freezing rate and storage temperature on muscle tissue before and after thawing. Adapted from [7,61,62].

Ice crystal formation is the defining physical process during seafood freezing and has a major influence on the final quality of frozen products. The size, number, and distribution of ice crystals are determined largely by the freezing rate and the stability of the storage temperature [59]. Slow freezing favors the formation of large extracellular ice crystals, which generate mechanical stress, rupture cell membranes, disrupt myofibrils, and damage connective tissues. In contrast, rapid freezing produces numerous small intracellular ice crystals that cause less structural damage and better preserve the native muscle architecture [6,24]. During frozen storage, ice crystals continue to change through recrystallization. Small crystals gradually dissolve while larger crystals grow, a process driven by Ostwald ripening. Temperature fluctuations, which commonly occur during transportation and retail distribution, accelerate this process and increase mechanical damage to muscle tissues [25,60]. As structural damage progresses, muscle proteins lose their ability to retain water, resulting in greater drip loss after thawing, softer texture, reduced firmness, and lower juiciness. Membrane disruption also releases free iron and endogenous enzymes that promote both lipid and protein oxidation, further accelerating quality deterioration [25,26]. In turn, oxidation weakens the protein network, making the muscle more susceptible to additional ice crystal damage during storage. This physical deterioration process is illustrated in Figure 4. Ice formation first concentrates solutes in the unfrozen phase, increasing ionic strength, protein concentration, pro-oxidant levels, and local pH changes (Figure 4A). The freezing rate then determines the extent of structural damage: rapid freezing produces smaller, more uniformly distributed ice crystals, whereas slow freezing forms larger crystals that cause greater cell disruption and protein denaturation (Figure 4B). During storage, repeated ice crystal growth and recrystallization progressively increase mechanical stress within the muscle (Figure 4C). Microscopic observations further demonstrate that slow freezing and temperature fluctuations lead to more severe disruption of muscle fibers and tissue structure after thawing (Figure 4D,E).

In summary, ice crystal formation and recrystallization are major contributors to structural damage in frozen seafood. Their effects on water retention, texture, and muscle integrity make them critical indicators of frozen storage quality and important targets for cold-chain quality monitoring.

### 2.5. Interplay of Deterioration Mechanisms

**Figure 5:**
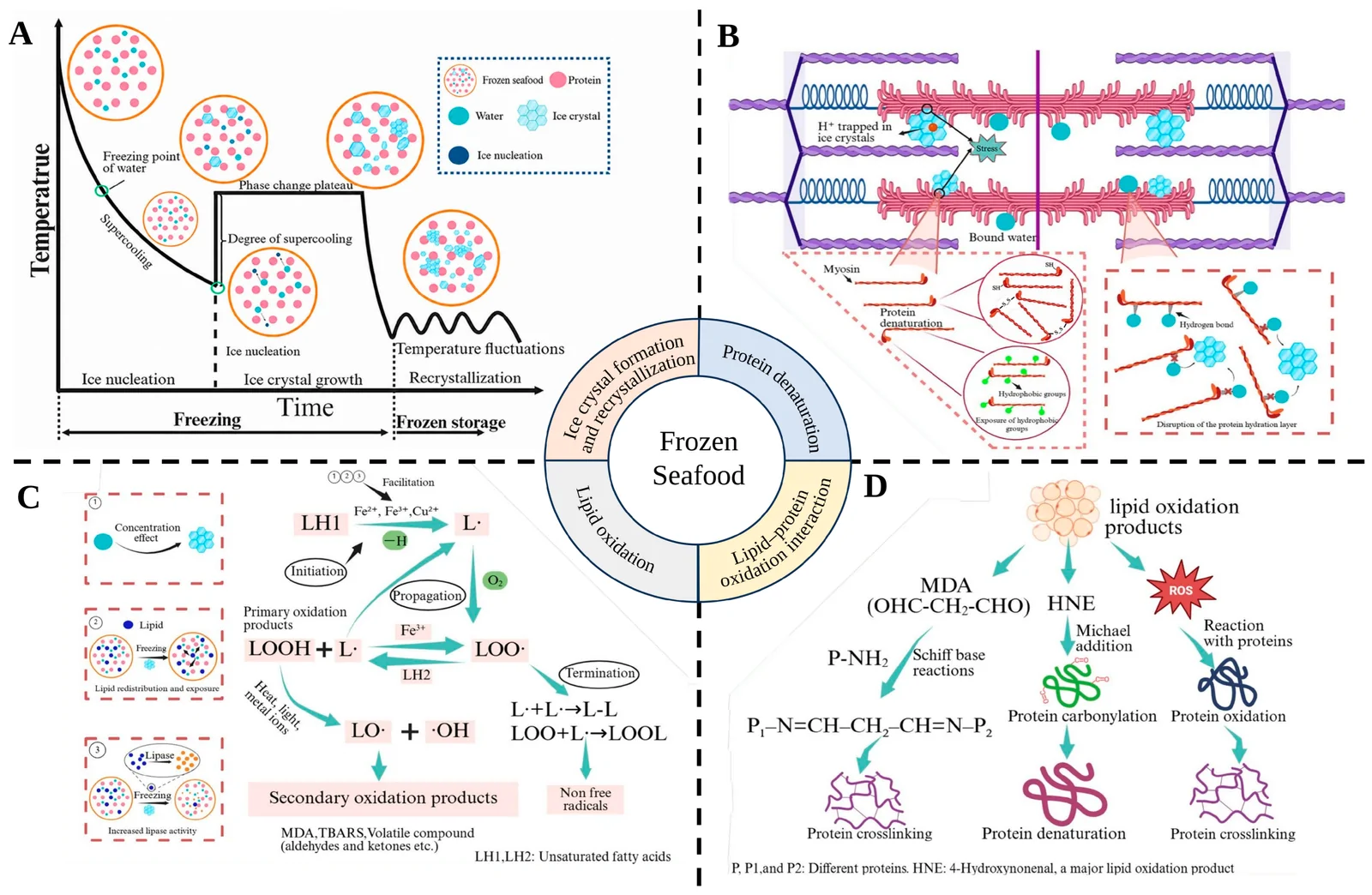
Integrated mechanisms of quality deterioration in frozen seafood. (**A**) Freezing initiates ice nucleation, ice crystal growth, recrystallization, freeze concentration, and temperature fluctuations. (**B**) Ice crystal formation disrupts hydration layers and promotes protein denaturation. (**C**) Lipid redistribution and exposure initiate lipid oxidation and the formation of primary and secondary oxidation products. (**D**) Reactive lipid oxidation products and reactive oxygen species promote protein oxidation, carbonylation, aggregation, and structural deterioration, illustrating the interconnected nature of quality loss during frozen storage. Adapted from [58].

The deterioration mechanisms described above do not occur independently but interact throughout freezing, frozen storage, and thawing, collectively accelerating quality loss [26,36]. Ice crystal-induced damage to cell membranes and muscle structure releases free iron, heme proteins, and endogenous enzymes into the muscle matrix. These pro-oxidative components promote both lipid and protein oxidation, amplifying quality deterioration beyond the direct effects of mechanical damage. The interaction between protein denaturation, protein oxidation, and lipid oxidation is particularly important in frozen seafood. Reactive lipid oxidation products, including malondialdehyde (MDA) and 4-hydroxynonenal (4-HNE), readily react with amino acid residues in myofibrillar proteins, promoting carbonyl formation, cross-linking, aggregation, and further structural deterioration accumulation, Schiff base and Michael adduct formation, cross-linking, and aggregation [25,42,63,64]. At the same time, protein denaturation weakens the muscle structure and reduces water-holding capacity, making the tissue more susceptible to ice crystal damage during subsequent freeze–thaw cycles. These interconnected processes also contribute to discoloration, off-flavor development, drip loss, and texture deterioration, leading to a gradual decline in consumer acceptance. Figure 5 summarizes these coupled deterioration pathways. Freezing initiates ice nucleation, ice crystal growth, recrystallization, freeze concentration, and temperature-fluctuation effects (Figure 5A). These physical changes destabilize muscle proteins by disrupting hydration layers and promoting protein denaturation (Figure 5B). Simultaneously, lipid redistribution and increased lipid exposure accelerate lipid oxidation and the formation of primary and secondary oxidation products (Figure 5C). Reactive aldehydes, including MDA and 4-HNE, then interact with proteins, promoting carbonylation, aggregation, and additional structural damage (Figure 5D). Together, these processes demonstrate that ice crystal damage, protein denaturation, and lipid oxidation function as closely connected rather than independent deterioration mechanisms.

The strong interaction among these quality changes highlights the limitations of relying on a single quality indicator, such as TBARS, TVB-N, or texture, to evaluate frozen seafood. Because each indicator reflects only part of the deterioration process, combining complementary sensing techniques provides a more complete assessment of product quality [65]. For example, Li et al. [20] demonstrated that integrating E-nose, E-tongue, and colorimeter data improved the prediction of K value, carbonyl content, TBARS, and Ca^2+^-ATPase activity in frozen horse mackerel, illustrating the advantages of multi-sensor data fusion for monitoring complex quality changes. Frozen seafood deterioration is a multidimensional process driven by the close interaction of ice crystal damage, protein denaturation, lipid oxidation, and water redistribution. This provides the scientific basis for using advanced sensing technologies together with multi-sensor data fusion to achieve more reliable quality assessment than conventional single-parameter methods.

## 3. Advances in Sensing Technologies for Frozen Seafood Quality Monitoring

**Figure 6:**
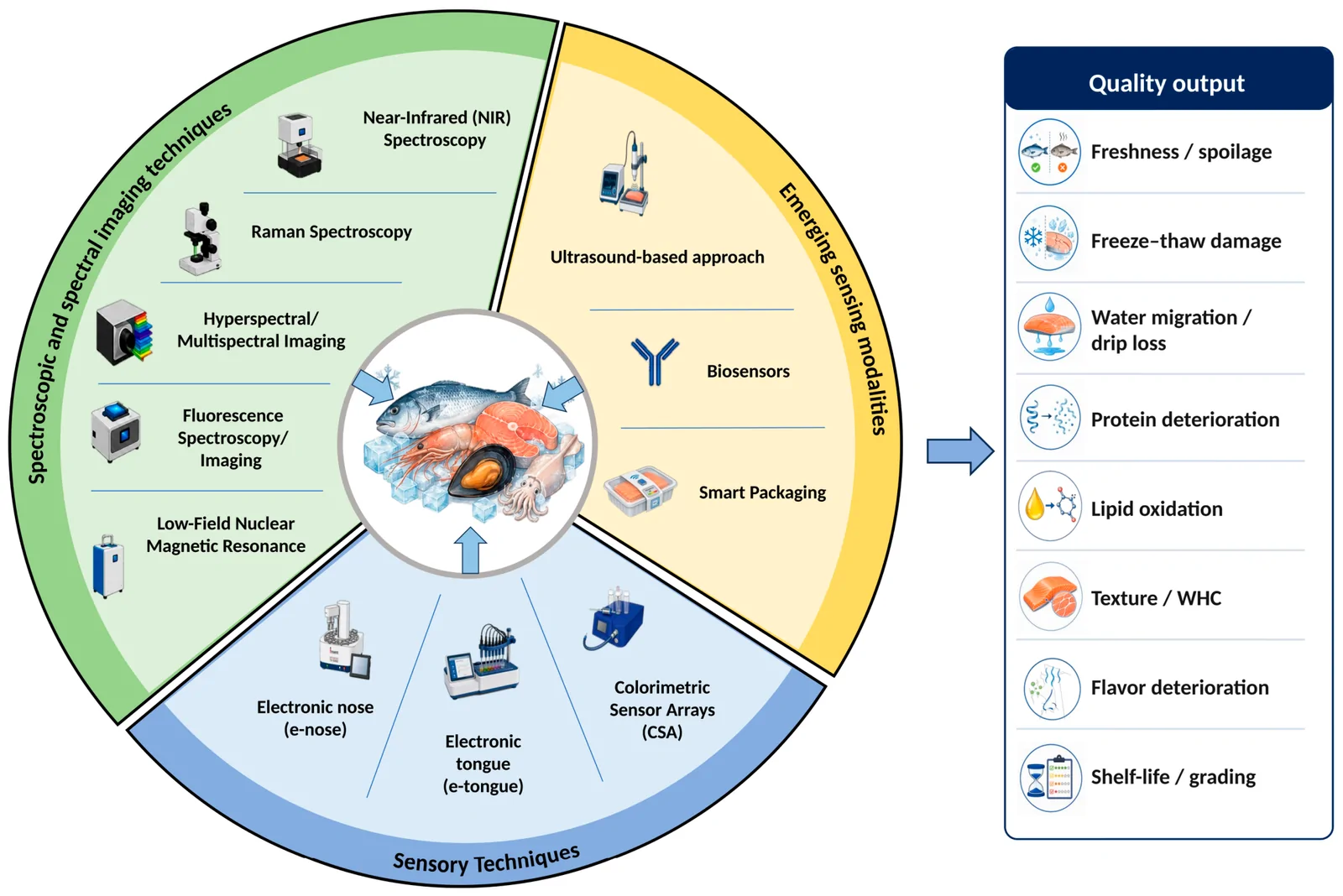
Overview of non-destructive sensing technologies used for frozen seafood quality assessment. Spectroscopic and spectral imaging techniques, sensory technologies, and emerging sensing modalities are linked to their major applications, including freshness evaluation, freeze–thaw detection, water migration, protein and lipid oxidation, texture assessment, flavor deterioration, shelf-life prediction, and quality grading.

Advanced sensing technologies have significantly expanded the tools available for evaluating frozen seafood quality, providing rapid, non-destructive alternatives to conventional laboratory methods. Because quality deterioration in frozen seafood is often complex, unevenly distributed, and difficult to detect visually, these technologies offer an effective way to monitor changes in molecular composition, water distribution, tissue structure, and freshness without damaging the product [66,67,68]. The major sensing technologies and the quality attributes they monitor are summarized in Figure 6. Spectroscopic and spectral imaging techniques are widely used to evaluate freshness, freeze–thaw damage, protein deterioration, lipid oxidation, and changes in water distribution. Electronic nose and electronic tongue mainly assess volatile compounds, odor, and taste-related quality changes, while emerging technologies, including smart packaging, colorimetric sensor arrays, biosensors, and time–temperature indicators, provide additional support for cold-chain monitoring, shelf-life prediction, and rapid quality screening. Together, these approaches provide complementary information for a more comprehensive assessment of frozen seafood quality.

### 3.1. Spectroscopic and Spectral Imaging Techniques

Spectroscopic and spectral imaging techniques are widely used for non-destructive food quality assessment because they provide molecular information and, in imaging-based systems, reveal the spatial distribution of quality changes across the sample surface [69,70]. In frozen seafood systems, these methods are especially effective for monitoring storage- and freeze–thaw-induced changes in proteins, lipids, and water distribution, the principal chemical drivers of quality deterioration [71,72].

#### 3.1.1. Near-Infrared (NIR) Spectroscopy

Near-infrared (NIR) spectroscopy measures the absorption of overtone and combination bands of C–H, O–H, and N–H functional groups within the 780–2500 nm spectral range. As these bonds are abundant in water, lipids, and proteins, NIR is well suited for evaluating the main quality components of seafood. In frozen seafood, it is commonly used to detect changes in water distribution, freeze–thaw history, freshness, and storage-related tissue alterations, as these changes influence the spectral response of muscle tissues [66].

**Figure 7:**
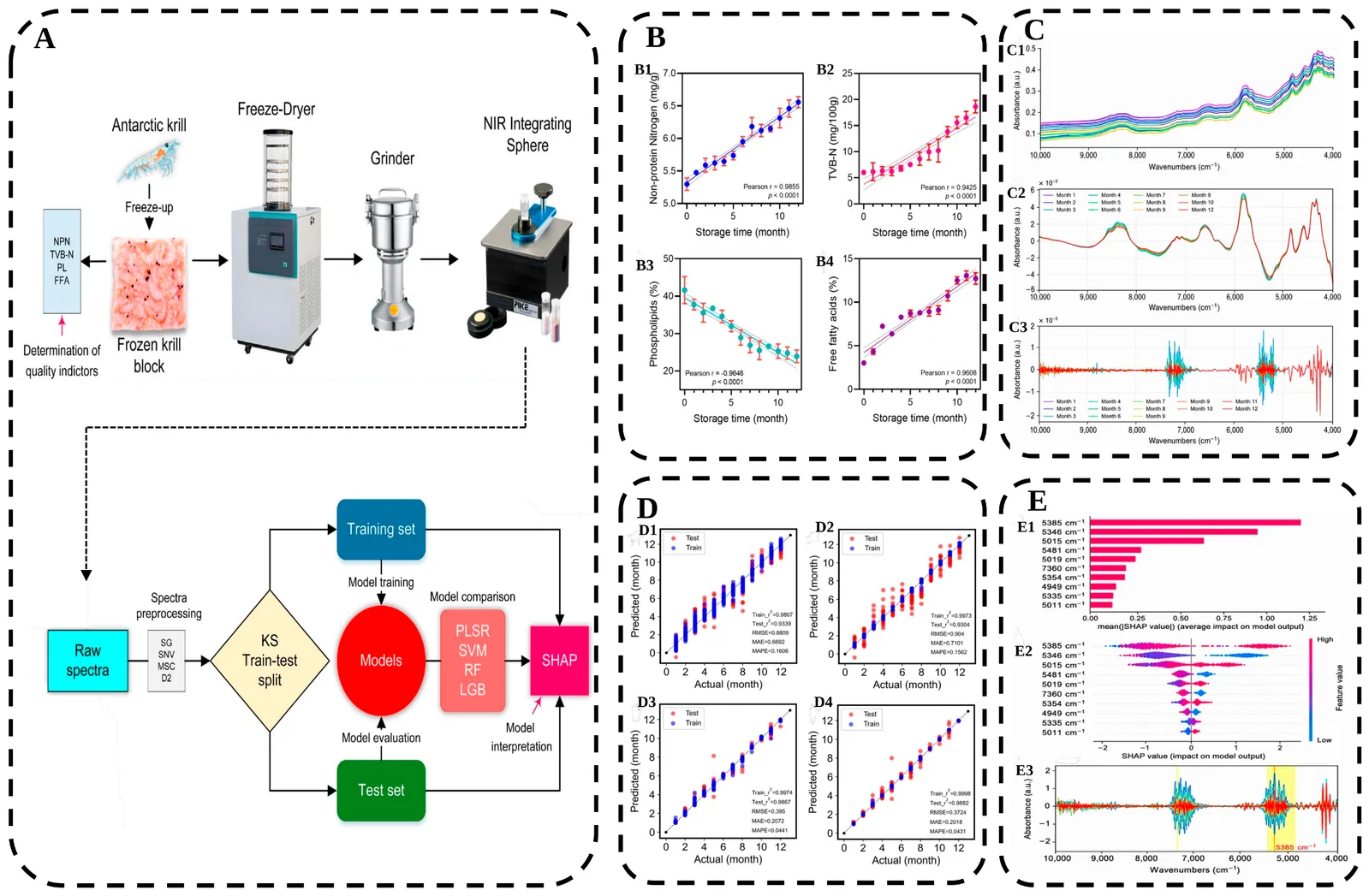
Representative NIR spectroscopy and machine learning workflow for frozen seafood quality prediction. (**A**) Sample preparation, NIR spectral acquisition, spectral preprocessing, dataset splitting, model development, and SHAP-based model interpretation. (**B**) Changes in reference quality indicators during frozen storage, including non-protein nitrogen, TVB-N, phospholipids, and free fatty acids. (**C**) Raw, preprocessed, and derivative NIR spectra used for model construction. (**D**) Prediction performance of different machine learning models for storage-related quality changes. (**E**) SHAP analysis identifying important wavelengths and their contribution to model outputs. Adapted from [73].

NIR spectroscopy has several practical advantages, including rapid analysis, minimal sample preparation, simple operation, and the availability of portable and at-line instruments. For example, Giró-Candanedo et al. [66] evaluated two low-cost NIR devices to distinguish fresh mackerel from samples subjected to one and two freeze–thaw cycles. The devices achieved classification rates of 90.3% and 94.1%, demonstrating the potential of NIR spectroscopy for identifying frozen–thawed fish marketed as fresh. However, performance declined when distinguishing different freezing systems after thawing, and seasonal and biological variability reduced model robustness. A representative NIR-based quality prediction workflow is presented in Figure 7. As illustrated in Figure 7A, the workflow includes sample preparation, spectral acquisition, preprocessing, dataset splitting, model development, and SHAP-based model interpretation. Figure 7B shows changes in reference quality indicators during frozen storage, including non-protein nitrogen, TVB-N, phospholipids, and free fatty acids. The corresponding raw, preprocessed, and derivative NIR spectra are presented in Figure 7C, while Figure 7D compares the performance of different machine learning models. Figure 7E highlights the wavelengths that contribute most to model predictions through SHAP analysis.

#### 3.1.2. Raman Spectroscopy

Raman spectroscopy is a vibrational technique that provides molecular fingerprint information through inelastic light scattering. Because water only has a weak Raman signal, this method is suitable for high-moisture foods such as seafood [74,75]. In frozen seafood, Raman spectroscopy has been used to monitor changes in protein secondary structures, lipid oxidation, and pigment states during freezing, frozen storage, and freeze–thaw cycles. Raman bands associated with myofibrillar proteins, unsaturated lipids, and myoglobin can provide useful information on texture deterioration, oxidative stability, and color changes [53,76]. For example, Guo et al. [53] combined.

NIR and Raman spectroscopy with a CNN model to predict TBARS values in salmon stored at static and dynamic temperatures. The fused model outcome of single-spectrum models, achieving $R_{p}^{2}$ = 0.866, RMSEP = 0.103 mg MDA/kg, and RPD = 4.136, demonstrated the value of complementary spectral information for monitoring lipid oxidation. Its practical limitations include the tradeoff between model complexity and predictive accuracy, as well as the lack of validation across different packaging types, which may restrict broader industrial application.

#### 3.1.3. Hyperspectral and Multispectral Imaging

Hyperspectral imaging (HSI) combines spectroscopy with spatial imaging by acquiring a complete spectrum for every pixel in a two-dimensional image, producing three-dimensional data cubes (x, y, λ) that contain both spatial and spectral information. This combination distinguishes HSI from point-based spectroscopic techniques and allows visualization of quality changes that develop unevenly across frozen seafood during storage [77,78].

HSI has been widely applied to frozen seafood quality assessment. It has been used to evaluate freshness indicators such as TVB-N and K value, monitor moisture migration, color and texture changes, identify freeze–thaw history, and visualize the spatial distribution of spoilage across seafood surfaces [77,78,79].

Yan et al. [79] used hyperspectral imaging of fish-eye information and deep learning to non-destructively determine after zero to freeze–thaw cycles in large yellow croaker; the spectral model achieved 87.5% accuracy, while fusion of spectral and eye-image features increased accuracy to 92.5%, showing that the two data types provided complementary information. However, the model still had difficulty distinguishing later freeze–thaw stages because the F2 and F3 samples showed similar changes.

**Figure 8:**
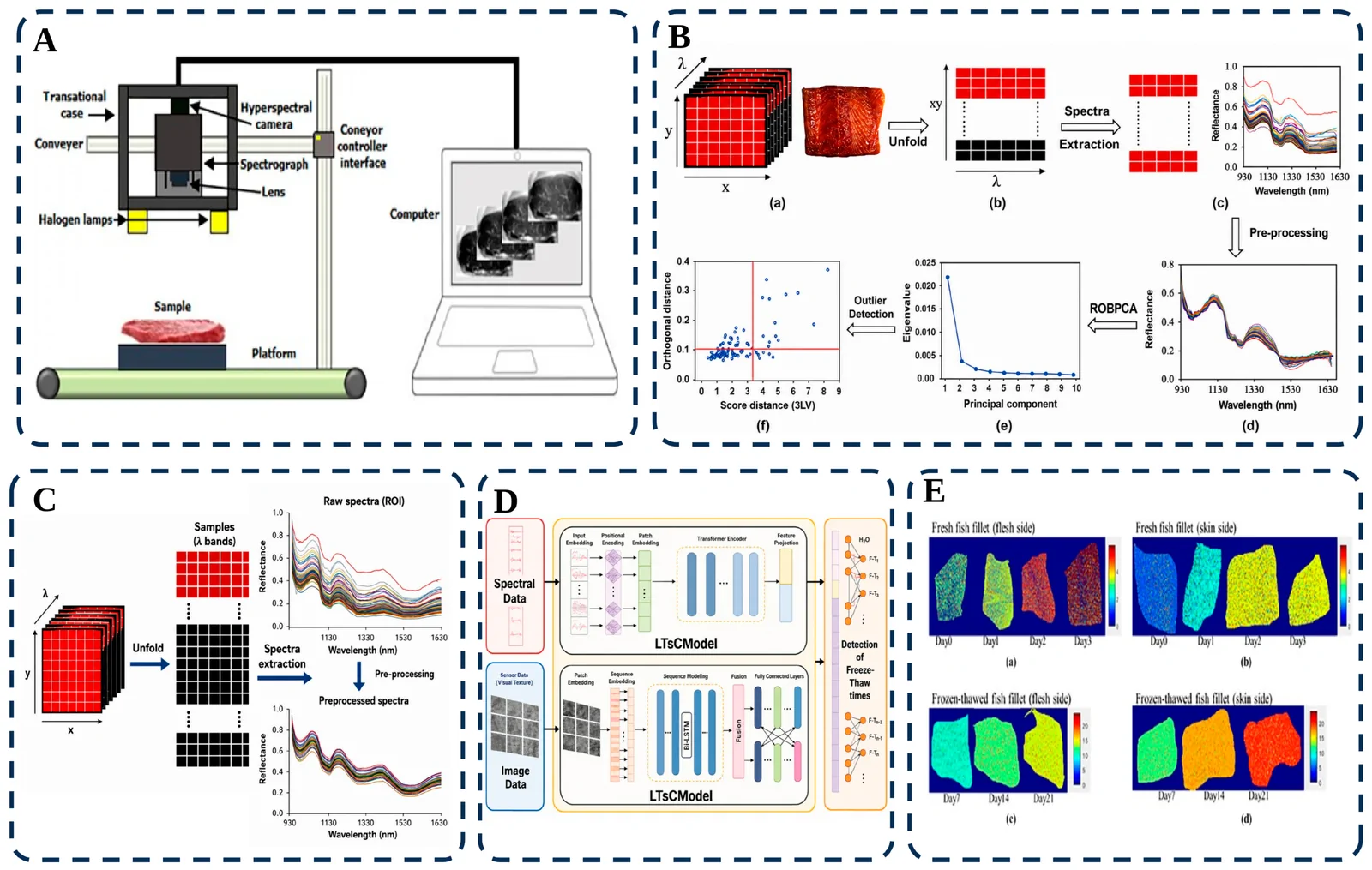
Hyperspectral imaging workflows and applications for seafood quality assessment. (**A**) Typical HSI acquisition system including a light source, conveyor platform, hyperspectral camera, spectrograph, and image collection system. (**B**) HSI data-processing workflow: (a) hyperspectral image cube and sample representation; (b) image-cube unfolding and spectral extraction; (c) raw spectra; (d) preprocessed spectra; (e) principal-component/eigenvalue analysis using ROBPCA; and (f) outlier detection based on score and orthogonal distances. (**C**) Spectral extraction and preprocessing from selected regions of interest. (**D**) Deep learning framework for detecting freeze–thaw history in fish products. (**E**) Representative visualization maps: (a) fresh fillet, flesh side; (b) fresh fillet, skin side; (c) frozen–thawed fillet, flesh side; and (d) frozen–thawed fillet, skin side. Adapted from [77,79].

Recent developments in HSI data analysis, including spectral–spatial feature extraction, machine learning, and deep learning, have further improved the ability to convert hyperspectral images into quantitative quality predictions and chemical distribution maps [78,79,80]. Multispectral imaging, which captures images at selected wavelengths, offers a more practical and cost-effective alternative for industrial applications while retaining the most informative spectral features. Figure 8 illustrates the main HSI workflow used in seafood quality assessment. Figure 8A shows a typical HSI acquisition system consisting of a light source, conveyor platform, hyperspectral camera, spectrograph, and image collection system. After image acquisition, hyperspectral cubes are processed through region-of-interest selection and spectral extraction. Figure 8B presents the complete processing workflow, including preprocessing, dimensionality reduction, and outlier detection, whereas Figure 8C shows spectral extraction and preprocessing from selected regions of interest. Figure 8D,E demonstrate a deep learning framework for freeze–thaw detection. Multispectral imaging, which acquires images at a limited number of pre-selected wavelengths, offers a cost-effective alternative to full HSI systems for industrial implementation while retaining spatial quality information at the most informative wavebands.

#### 3.1.4. Fluorescence Spectroscopy and Imaging

Fluorescence spectroscopy measures the natural fluorescence emitted by endogenous food components, including aromatic amino acids such as tryptophan and tyrosine, NADH, porphyrins, vitamins, and lipid oxidation products such as Schiff bases. The excitation–emission matrix (EEM) provides a two-dimensional fluorescence fingerprint that reflects protein conformational changes, oxidative degradation, and pigment transitions during frozen storage and freeze–thaw cycles [16,68]. Rahman et al. [68] demonstrated that fluorescence fingerprints evaluate the pre-freezing freshness of frozen spotted mackerel without thawing. The method clearly showed the differences between white and dark muscle and predicted the K-value more accurately in white meat $R^{2}=0.87$ than in dark meat $R^{2}=0.82$. However, prediction of pH and IMP was much weaker in dark muscle. The authors also emphasized the need for longer postmortem storage periods, larger sample sets, validation in different value-added fish products, and a smaller portable fluorescence spectrometer.

#### 3.1.5. Low-Field Nuclear Magnetic Resonance

Low-field nuclear magnetic resonance (LF-NMR) directly measures the distribution and mobility of water within muscle tissue, making it a valuable non-destructive technique for frozen seafood quality assessment [15,81,82]. By measuring proton transverse relaxation times (T_2_) using the Carr–Purcell–Meiboom–Gill (CPMG) pulse sequence, LF-NMR distinguishes different water populations, including tightly bound water (T_2_b, ~0.1–10 ms), immobilized water within the myofibrillar protein network (T_21_, ~10–100 ms), and free water in the extracellular space (T_22_, >100 ms) [83,84].

**Figure 9:**
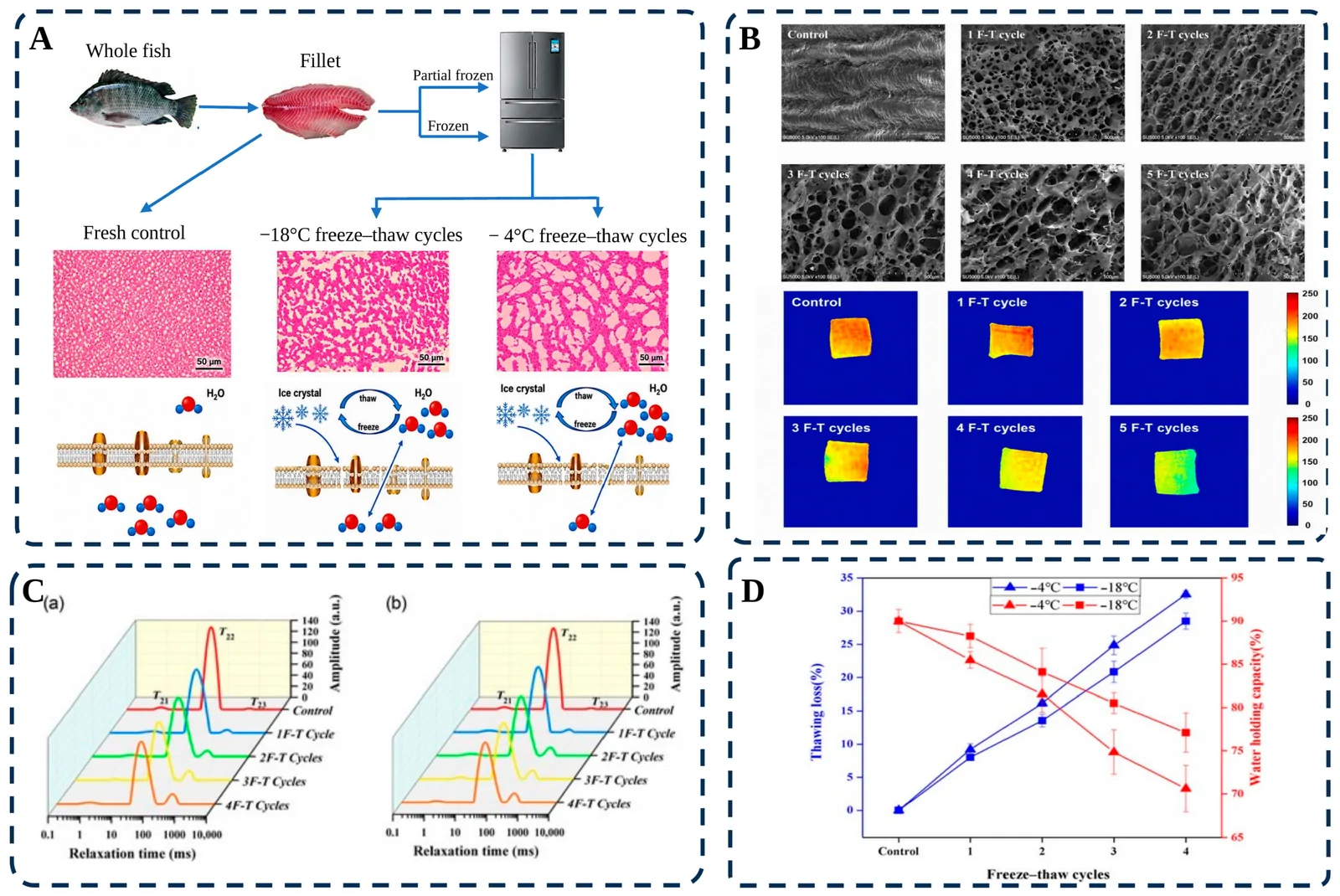
Freeze–thaw-induced water migration and structural deterioration in frozen fish muscle. (**A**) Experimental workflow showing fish processing, filet preparation, freezing or partial freezing, and quality analysis. (**B**) Microscopic evidence of progressive tissue damage during repeated freeze–thaw cycles. (**C**) (a,b) T_2_ relaxation-time distribution curves of tilapia fillets subjected to different numbers of freeze–thaw cycles (FTCs) at −4 and −18 °C. (**D**) Repeated freeze–thaw cycles increase thawing loss and reduce water-holding capacity, indicating progressive deterioration of muscle structure and water retention. Adapted from [81,86].

During frozen storage and repeated freeze–thaw cycles, protein denaturation and muscle damage cause water to shift from the immobilized fraction to the free-water fraction. These changes appear as shifts in LF-NMR relaxation signals and are commonly associated with increased drip loss and reduced texture quality [81,85]. Figure 9 illustrates the relationship between freeze–thaw treatment, water migration, microstructural damage, and water-holding capacity. Figure 9A shows the experimental workflow, including fish processing, filet preparation, freezing or partial freezing, and quality evaluation. Progressive tissue damage after repeated freeze–thaw cycles is shown in Figure 9B, while Figure 9C presents LF-NMR relaxation profiles corresponding to bound, immobilized, and free-water fractions. The resulting water redistribution is reflected by increased thawing loss and reduced water-holding capacity in Figure 9D. Luo et al. [81] reported that LF-NMR relaxation parameters were closely associated with water-holding capacity, drip loss, and texture changes in frozen fish, demonstrating the usefulness of LF-NMR for monitoring internal water migration and structural deterioration.

### 3.2. Sensor Arrays for Volatile, Taste, and Gas Detection

#### 3.2.1. Electronic Nose (E-Nose)

Electronic nose (E-nose) systems consist of arrays of gas sensors, commonly metal oxide semiconductor (MOS), conductive polymer, or quartz crystal microbalance sensors, combined with pattern recognition algorithms. During frozen storage and thawing, seafood releases a changing mixture of volatile organic compounds (VOCs), including aldehydes, ketones, alcohols, sulfur-containing compounds, and biogenic amines, which are mainly produced by lipid oxidation, protein degradation, and microbial activity [20,87].

E-nose systems have been widely used to distinguish frozen storage periods and classify freshness in frozen fish. Changes in the response patterns of the sensor array reflect the accumulation of VOCs during storage, allowing rapid freshness evaluation and semi-quantitative estimation of shelf life [20]. For example, Li et al. [20] combined E-nose, E-tongue, and colorimeter data with machine learning models to predict the freshness of horse mackerel stored at −18 °C for 90 days. The integrated sensing approach improved the prediction of K value, carbonyl content, TBA, and ${Ca}^{2+}$-ATPase activity, with $R_{p}^{2}$ values of 0.936–0.994, demonstrating the advantages of combining multiple sensing techniques for frozen seafood quality assessment. A key limitation is that the method required partial thawing and mincing. Moreover, the models were developed from only 30 sensor observations using an internal 7:3 split, without independent external validation.

#### 3.2.2. Electronic Tongue (E-Tongue)

Electronic tongue (E-tongue) systems use arrays of electrochemical sensors, including potentiometric, amperometric, and voltammetric sensors, to detect taste-related dissolved compounds such as free amino acids, nucleotides, organic acids, salts, and bitter substances in seafood extracts [88]. In frozen seafood, protein denaturation and proteolysis during storage and thawing, ATP degradation through the IMP–inosine–hypoxanthine pathway, and oxidative reactions alter the composition of these taste-active compounds, allowing E-tongue systems to assess quality changes rapidly and objectively [20,89]. For example, Fan et al. [89] used an E-tongue to evaluate taste changes in the gonads of female Chinese mitten crab stored at −20, −40, and −80 °C. PCA explained 98.06% of the variance and produced a discrimination index of 96, indicating clear separation among storage conditions. A methodological limitation was that no established Hx threshold was available, so its fuzzy membership ranges were defined from the experimental results. Moreover, the model was applicable for only frozen gonads and would require new taste indicators and membership functions for fresh samples.

#### 3.2.3. Colorimetric Sensor Arrays (CSAs)

Colorimetric sensor arrays (CSAs) use different dye materials, including metal–porphyrin complexes, pH-sensitive dyes, and redox-responsive compounds, immobilized on a solid substrate. These dyes change color when they interact with volatile spoilage compounds such as amines, sulfur compounds, aldehydes, and organic acids [90,91,92]. In frozen seafood, CSAs are most useful during thawing and packaged storage, when volatile amines and other spoilage-related gases become more abundant. They provide a simple visual or digital indication of freshness and can be incorporated into smart packaging systems [91,93,94].

Recent advances in dye chemistry, printable substrates, and camera-based color analysis have improved the sensitivity and practicality of CSA-based freshness indicators. For example, Baptista et al. [91] developed a polysaccharide-based screen-printed colorimetric sensor for detecting amines in packaged fish, demonstrating the potential of on-package color indicators for monitoring fish spoilage. Ameri et al. [93] further showed a black rice anthocyanin on-package indicator for pangasius filets stored at 4 °C. The indicator provided four visible freshness stages, and its color response correlated strongly with TVB-N $R^{2}=0.9836$ and pH $R^{2}=0.9878$. Its main weakness was that the color response plateaued at high spoilage levels, reducing its ability to differentiate advanced spoilage stages.

### 3.3. Emerging Sensing Modalities

Biosensors combine biological recognition elements, such as enzymes, antibodies, aptamers, or nucleic acids, with signal transducers to detect specific freshness- or safety-related compounds. Compared with spectroscopic and imaging techniques, their application in frozen seafood is still limited. Early studies showed that biosensors could detect biochemical quality indicators in refrigerated and frozen sea bass, suggesting their potential for fish quality assessment under cold-storage conditions [95]. More recent studies have focused on freshness biomarkers, including hypoxanthine, histamine, and biogenic amines, which are better suited for targeted post-thaw inspection than continuous monitoring of frozen products [96]. At present, biosensors are more suitable for detecting specific biomarkers than for routine quality assessment throughout the frozen seafood cold chain.

Ultrasound-based approaches have also attracted attention for assessing the internal structure of frozen seafood. Ultrasonic wave propagation is affected by tissue density, elasticity, water distribution, and microstructure, making it useful for detecting internal quality changes that are difficult to identify from surface appearance alone [97,98]. These features make ultrasound potentially useful for detecting internal quality differences that are difficult to evaluate from surface appearance alone [99,100,101]. For example, Yagi et al. [102] combined ultrasound inspection with machine learning to evaluate frozen albacore tuna. Their model achieved 84.2% accuracy in identifying fat-rich albacore, compared with 73.6% using the conventional tail-cutting method, demonstrating the potential of ultrasound for non-destructive quality grading. The study was limited by its relatively small dataset of 57 fish and the manual acquisition of signals from 24 probe positions per sample. The authors therefore emphasized the need for larger datasets and automated batch inspection or robotic systems before industrial application.

Smart packaging is becoming an important tool for monitoring frozen seafood during cold-chain distribution, particularly through time–temperature indicators (TTIs), freshness indicators, sensor-based labels, and RFID-assisted tracking systems. Tsironi et al. [103] carried out a pilot study using frozen blue shark slices and arrow squid under commercial cold-chain conditions from production to consumption. UV-activatable and enzymatic TTIs were attached to the products to monitor shelf life during frozen distribution. The indicators translated cumulative temperature exposure into product-specific shelf-life information instead of relying only on fixed expiry dates. A key limitation is that each TTI requires validated product-specific deterioration models and careful matching between the activation energy of the indicator and that of the selected quality index. More recent studies and reviews have also shown that freshness indicators, TTIs, amine-responsive labels, and digital tracking systems can support seafood quality and shelf-life monitoring, although validation under frozen seafood cold-chain conditions is still relatively limited [91,93,104,105].

Table 1 compares the main non-destructive sensing technologies used for frozen seafood quality assessment. Spectroscopic and imaging techniques, including NIR, Raman spectroscopy, HSI/MSI, fluorescence imaging, and LF-NMR, provide information on molecular composition, spatial variation, and water distribution associated with quality deterioration. Sensory and emerging approaches, such as E-nose, E-tongue, colorimetric sensor arrays, biosensors, smart packaging, and ultrasound, provide complementary information on volatile compounds, taste changes, freshness indicators, cold-chain history, and internal structural quality. Since no single technique can capture every aspect of frozen seafood quality, the choice of method depends on the target quality attribute, detection accuracy, measurement speed, cost, portability, industrial readiness, and practical limitations.

**Figure 10:**
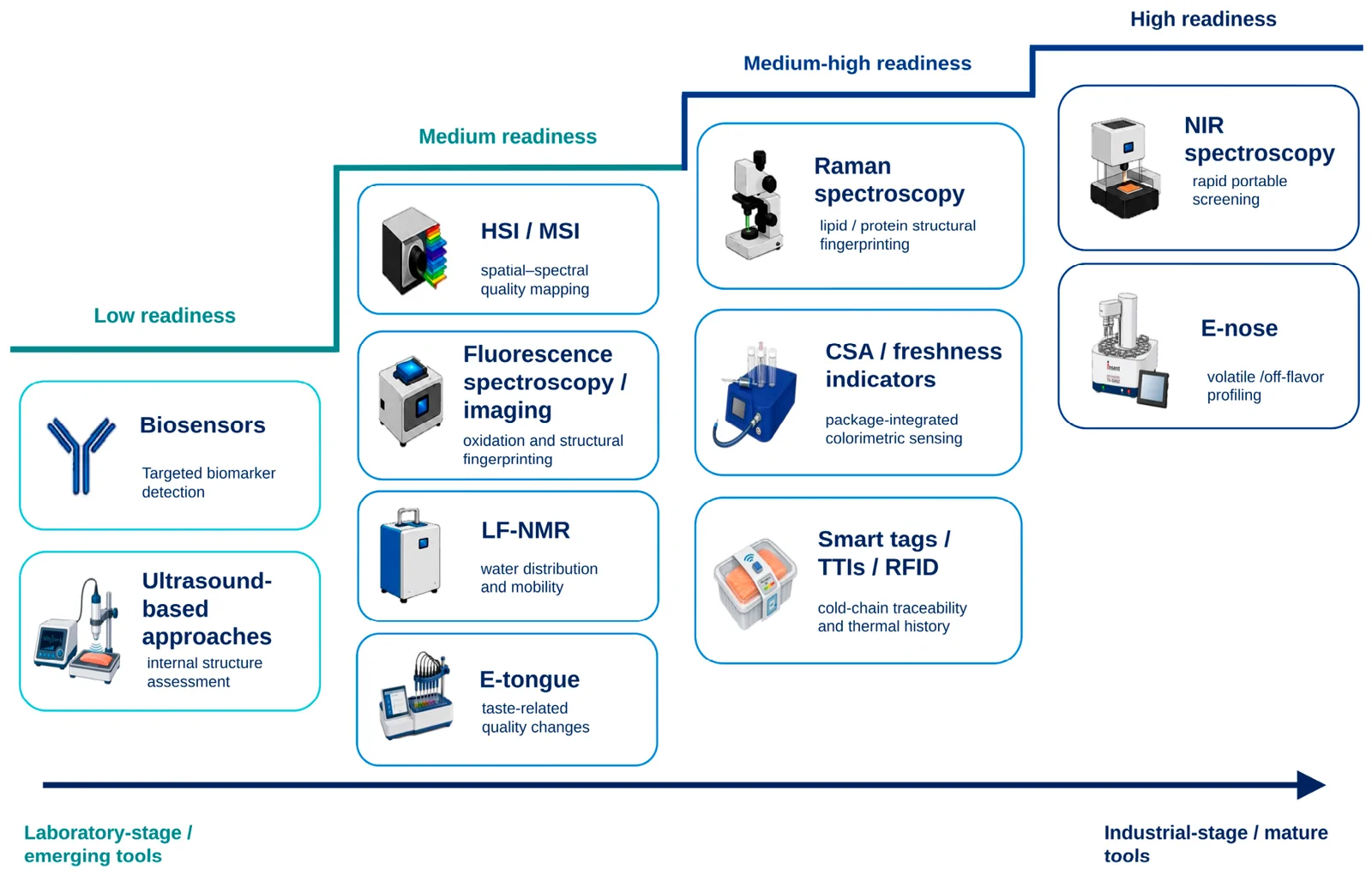
Technology readiness of sensing modalities for frozen seafood quality assessment. Technologies are ranked according to validation evidence, portability, industrial feasibility, and cold-chain suitability, from emerging laboratory approaches to mature methods for industrial application.

The sensing technologies summarized in Table 1 differ not only in the quality indicators they target and their analytical capabilities but also in their readiness for industrial application. Figure 10 compares these technologies based on validation evidence, portability, industrial feasibility, and suitability for frozen seafood cold-chain monitoring, providing an overview of their current level of practical implementation.

### 3.4. Laboratory to Industry: Cost, Robustness, and Regulatory Challenges

In addition to the analytical performance, the usage of these sensing technologies in the industrial field depends on several practical factors, including calibration stability, cost, maintenance, measurement speed, standardization, and ease of operation.

Calibration transfer and sensor robustness. Methods such as NIR and Raman spectroscopy often face calibration transfer problems; a model that is trained on one instrument may not generalize well to another instrument with the same type because of variations in optical components and detectors. It requires recalibration whenever hardware is replaced or deployed to deferent production sites [111,112,113]. Beyond instrument-level recalibration, there is currently no industry-wide standard comparable to AOAC or ISO reference methods for validating any of these sensing technologies against frozen seafood quality endpoints, which means each research group effectively defines its own calibration and validation criteria [114]. HSI has additional calibration challenges because differences in lighting and camera position between sites can affect the image quality and reduce the accuracy of the model [114]. LF-NMR is comparatively more stable once calibrated, but it requires temperature control to maintain accuracy [115]. Sensor array systems face a related issue, which is sensor drift. The responses of MOS and electrochemical sensors may change with repeated use, humidity, and environmental exposure. Without regular recalibration or drift correction, this can reduce classification accuracy [116,117]. Biosensors also have stability problems because enzymes and antibodies can lose activity over time and must be stored under controlled conditions [118,119]. The challenge of ultrasound is that the probe must maintain consistent contact with the sample, which can be difficult to reproduce across operators [120,121]. Smart packaging indicators are stable during use since TTIs and colorimetric labels do not require recalibration after activation. Species and different products create another challenge [103,104]. Models that are developed for one kind of seafood species, muscle type, or product may not perform well when they are applied to another because of water content, lipid composition, muscle structure, and volatile profiles of the products [66].

Cost and maintenance. Portable NIR and colorimetric sensor arrays are generally closer to industrial use because they are affordable, easy to operate, and suitable for measurements outside the laboratory. HSI, Raman spectroscopy, and LF-NMR usually require more expensive equipment and require more controlled operating environments. Their use is therefore concentrated mainly in research laboratories or pilot-scale applications. E-nose and E-tongue systems are in the middle in terms of cost, but the sensor arrays gradually wear out and need replacement. Biosensors have similar ongoing costs because their enzyme and antibody components have a limited shelf life and must be replaced regularly. Ultrasound equipment may cost about the same as NIR systems, but its cost-performance profile for frozen seafood specifically is still not well established. The equipment cost is not the only concern; sensor replacement, cleaning, recalibrations, and software maintenance can create additional expenses. Beyond the equipment itself, sensor and enzyme replacement for E-nose, E-tongue, and biosensor systems is typically a simple part swap, while HSI and Raman systems require optical realignment and lens cleaning to maintain measurement accuracy, usually performed by trained technicians rather than production line staff. So, maintenance adds ongoing labor costs beyond the sensor replacement itself.

Online monitoring. Readiness for continuous, in-line monitoring is very different across these technologies, and little of it has been tested on real production lines. NIR and colorimetric sensors are the most suitable for in-line or at-line use because they are fast and use simple optics [66,93]. Raman spectroscopy is slower to adapt to line speed because of its weak signal and longer acquisition time [74]. HSI requires a large amount of data processing, which currently limits real-time use [77]. LF-NMR depends on a stable magnetic field, which makes in-line integration difficult with current hardware [122]. E-nose and E-tongue systems are portable but usually need discrete sampling and short equilibration time, so they are better suited for at-line spot-checks than continuous inspection [20,89]. Biosensors currently target specific biomarkers for post-thaw inspection rather than continuous monitoring [96]. Ultrasound-based methods are promising for internal quality grading, but they still need more validation before they can be used at line speed [121]. Smart packaging is the exception: because TTIs, freshness labels, and RFID tracking are built into the package itself instead of needing a separate instrument, they are naturally suited to continuous, real-time cold-chain monitoring, although standardized validation for frozen seafood specifically is still limited [103,104]. This gap between laboratory testing and continuous production monitoring is one of the clearest barriers to industrial use across the field.

Regulatory approval and standardization. At present, regulatory acceptance of advanced sensing technologies for frozen seafood remains limited, particularly for quality and fraud determination comparable to wet-chemistry methods such as TVB-N or TBARS. Part of the barrier is procedural: in the United States, official recognition typically requires validation against AOAC International’s Official Methods of Analysis, while in the EU, food-fraud control methods fall under Regulation (EU) 2017/625 [123], and neither framework has an approved pathway specific to sensor- or model-based non-destructive methods for seafood yet. NIR has been formally reviewed in this regulatory context for other food categories, such as nuts [124], but no comparable regulatory pathway currently exists for frozen seafood sensing technologies. Because these methods rely on chemometric or machine learning models rather than a single fixed chemical threshold, validating them against existing regulatory frameworks, which were built around fixed endpoint reference methods, it is a challenge by itself. As a result, these sensing methods are used today as screening tools rather than replacements for laboratory testing.

Operator training and commercialization. Even when instruments are affordable and portable, their results often depend on chemometric or machine learning models that typically require standardized sample presentation or operating protocols, which is difficult to distinguish from genuine quality differences [125]. Portable NIR systems and colorimetric indicators are the most commercially mature and have shown good potential in seafood quality assessment [125]. This is reflected in the instrument market itself: several established manufacturers (e.g., Thermo Fisher Scientific, VIAVI Solutions) now sell handheld and miniaturized NIR units marketed for on-site food quality screening, whereas no equivalent commercial product exists yet for HSI, Raman, or LF-NMR systems specifically designed for frozen seafood. Smart packaging tools such as TTIs and RFID tags are among the most commercially established technologies reviewed here and already deployed in real cold-chain logistics [103,104]. E-nose and E-tongue systems are commercially available but remain largely confined to quality-control laboratories rather than production lines [126]. HSI, Raman spectroscopy, LF-NMR, biosensor-based systems, and ultrasound-based methods remain predominantly at the research and pilot-scale technologies [114,121,125]; the absence of dedicated commercial product lines for these technologies, in contrast to NIR, is itself evidence of how far they remain from routine industrial deployment. This suggests that further validation and standardization are needed before wider industrial application.

## 4. Data Integration and Artificial Intelligence for Quality Prediction

Artificial intelligence (AI) has become an important tool for analyzing the large datasets generated by advanced sensing technologies, helping convert them into reliable predictions, classifications, and quality assessments [73,127]. In frozen seafood, AI is applied to spectroscopic, imaging, and sensor data, either individually or in combination, to predict physicochemical quality indicators, classify freshness, detect quality changes, and estimate shelf life. Depending on the application, different approaches can be used, ranging from classical chemometric methods and machine learning to deep learning and explainable AI.

### 4.1. Chemometrics and Classical Machine Learning

Chemometric methods are widely used to analyze the large amount of data generated by spectroscopic and sensor-based techniques. Data obtained from NIR spectroscopy, Raman spectroscopy, HSI, NMR, E-nose, and E-tongue are often high-dimensional and highly correlated, making direct interpretation difficult. Chemometric analysis helps simplify these datasets and identify relationships between sensing signals and seafood quality attributes [66,67].

Principal component analysis (PCA) is one of the most common tools used to explore spectral datasets. It reduces data complexity, making it easier to visualize sample groupings, detect outliers, and examine sources of variation. In frozen seafood studies, PCA has been used to distinguish fresh and frozen–thawed fish and to investigate clustering patterns in spectral and NMR data. For example, Kaltenbach et al. [67] combined NMR with multivariate analysis to differentiate fresh fish from frozen–thawed samples. Their results showed that multivariate analysis could detect freeze–thaw differences that were difficult to identify from the raw data alone. Since PCA is an unsupervised method, it is generally used together with supervised models when quantitative prediction is required. Partial least squares regression (PLSR) and partial least squares discriminant analysis (PLS-DA) are among the most frequently used chemometric methods for prediction and classification. They are well suited to spectral data because they can handle highly correlated variables while linking spectral information with reference quality measurements. In frozen seafood, Giró-Candanedo et al. [66] used a portable NIR device together with PLS-DA to distinguish fresh mackerel from fish that had undergone one or two freeze–thaw cycles. This study showed that chemometric models can help identify frozen–thawed fish that could otherwise be sold as fresh.

Classical machine learning methods, including support vector machines (SVMs), random forest (RF), extreme gradient boosting (XGBoost), and artificial neural networks (ANNs), are increasingly used to model the complex relationships found in seafood quality data. Li et al. [20] combined E-nose, E-tongue, and colorimeter measurements with ANN, XGBoost, random forest regression, and support vector regression models to predict freshness indicators in horse mackerel stored for 90 days under frozen conditions. Using data from multiple sensors improved the prediction of K value, lipid oxidation, protein oxidation, and protein denaturation. Feature selection methods such as competitive adaptive reweighted sampling (CARS), genetic algorithms (GAs), and the successive projections algorithm (SPA) can further improve model performance by selecting the most informative wavelengths or sensor signals while reducing unnecessary variables.

Careful model validation is essential before these methods can be applied in practice. Cross-validation, independent test sets, and external validation using samples collected from different instruments, batches, or storage facilities help ensure that prediction models remain reliable under real operating conditions [128,129]. Without proper validation, models that perform well in the laboratory may not achieve the same level of accuracy when applied to commercial seafood products. Classical chemometrics and machine learning remain important tools for analyzing spectral and sensor data in frozen seafood quality assessment. They offer a good balance between prediction accuracy, model interpretability, and practical application, making them well suited for many current quality monitoring tasks.

### 4.2. Deep Learning for Feature Extraction and Quality Modeling

Deep learning approaches, especially convolutional neural networks (CNNs), are now widely used to analyze image-rich and high-dimensional sensing datasets in frozen seafood quality assessment. Unlike classical methods that rely on manually selected features and predefined statistical relationships, deep learning models learn useful features directly from raw images, spectral–spatial data, and sensor inputs. This allows them to recognize visual, textural, and structural patterns associated with freshness loss, freeze–thaw damage, and other quality changes. In frozen seafood quality monitoring, CNN-based and hybrid deep learning models have been applied to freshness grading, quality classification, and freeze–thaw cycle detection. For example, Genç et al. [127] evaluated several machine learning and deep learning models for classifying the freshness of frozen–thawed shrimp during refrigerated storage, highlighting the value of explainable AI for seafood quality assessment. Yan et al. [79] also combined hyperspectral imaging of fish-eye information with a cross-modal deep learning approach to determine freeze–thaw cycles in large yellow croaker. Their findings showed that deep learning could detect hidden temperature-abuse history that is difficult to identify using conventional methods.

Beyond CNNs, hybrid models such as CNN–LSTM combine spatial feature extraction with temporal or sequential information, making them useful for monitoring quality changes that occur gradually during frozen storage. Explainable AI can further improve model transparency by showing which image regions, spectral features, or sensor variables contribute most to prediction results. This makes model outputs easier to understand and provides useful support for freshness grading, freeze–thaw detection, and quality control [130].

Deep learning models still have some limitations. They often require large, annotated datasets, careful validation, and substantial computational resources. They may also overfit small laboratory datasets and perform less consistently across different species, batches, instruments, storage conditions, or processing facilities. For this reason, future studies should include external validation, explainability analysis, and testing under realistic cold-chain conditions. These validation issues are well recognized in spectral data modeling and are essential for translating deep learning models from laboratory studies into practical quality-control applications [128].

Deep learning has become an important tool for analyzing complex sensing data in frozen seafood quality assessment, particularly for image-based freshness classification, hyperspectral detection of freeze–thaw cycles, and other high-dimensional sensing applications. Its successful application, however, depends on reliable validation, interpretable models, and consistent performance under practical cold-chain conditions.

### 4.3. Multi-Sensor Data Fusion

**Figure 11:**
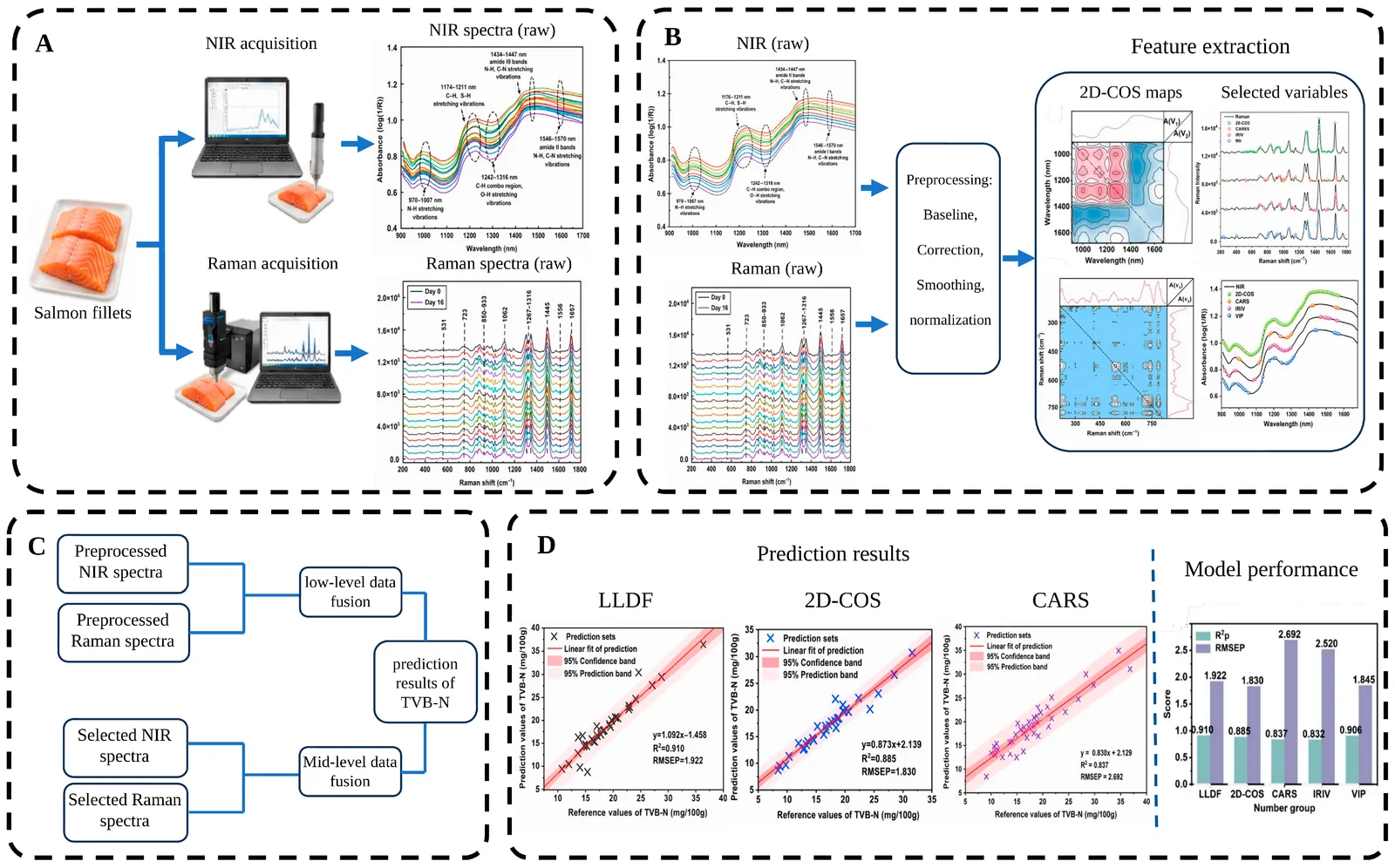
NIR–Raman spectral data fusion workflow for frozen seafood quality prediction. (**A**) Acquisition of raw NIR and Raman spectra from salmon fillets. (**B**) Spectral preprocessing and feature extraction, including 2D-COS analysis and selection of informative spectral variables. (**C**) Low-level fusion of preprocessed NIR and Raman spectra and mid-level fusion of selected spectral features for TVB-N prediction. (**D**) Prediction results obtained using different data-fusion strategies and comparison of model performance. Adapted from [54].

Multi-sensor data fusion strategies integrate complementary information from multiple sensing modalities to achieve quality predictions that are complete and more robust than those obtained from a single sensor. Three fusion architectures are commonly recognized: low-level fusion, which combines raw or preprocessed signals from different sensors into one feature matrix before modeling; mid-level fusion, which extracts key features from each modality before integration; and high-level fusion, which combines the outputs of separate modality-specific models through ensemble strategies [19]. In frozen seafood applications, data fusion has shown clear improvements in prediction performance. Guo et al. [53] reported that combining NIR and Raman spectra improved the non-destructive prediction of TBARS values in salmon flesh compared with single-modality models because the two techniques provide complementary information on water, lipids, and molecular structural changes. Similarly, Guo et al. [54] found that NIR–Raman fusion improved the prediction of TVB-N in salmon filets stored under different temperature conditions, highlighting the value of multimodal spectroscopy for freshness assessment in frozen and temperature-fluctuated seafood systems. This NIR–Raman fusion strategy is illustrated in Figure 11, where NIR and Raman spectra are first collected and preprocessed separately before being combined through low-level fusion of spectral variables and mid-level fusion of selected informative features. The fused spectral data are then used to develop prediction models for quality indicators such as TVB-N, showing how the combination of chemical and structural information can improve frozen seafood quality assessment.

However, multi-sensor data fusion also increases data complexity. Different sensing techniques generate signals with different scales, dimensions, noise levels, and acquisition conditions, making data integration more challenging. As a result, inappropriate fusion strategies may increase model complexity without improving prediction performance. Future studies should therefore focus not only on improving prediction accuracy, but also on selecting complementary sensors, reducing redundant variables, improving model interpretability, and validating fusion models under practical cold-chain conditions [131].

Multi-sensor data fusion remains an important approach for frozen seafood quality assessment. By combining information from different sensing technologies, it can improve the detection of lipid oxidation, freshness loss, color changes, volatile compounds, and other storage-related quality changes. Its successful application, however, depends on practical model design, reliable validation, and suitability for industrial use.

Beyond the methods discussed above, several emerging AI approaches are starting to be used in food quality assessment, although they have not yet been applied to frozen seafood specifically. Vision transformers are used for grading food quality from images, achieving high accuracy while running efficiently on edge devices, unlike CNNs, which use local filters; ViTs use self-attention to capture spatial relationships across the whole image, which could help detect spread-out spoilage or freeze–thaw damage rather than just localized defects. [132]. This has not been tested on frozen seafood yet, but it is a natural extension of image-based quality grading.

Foundation models and multimodal transformers combine images, spectra, and sensor data using attention mechanisms, and they have been used in food safety systems for fast and real-time results. Foundation models are pretrained on large, diverse datasets and then fine-tuned with comparatively little new data, rather than trained separately for each instrument or species, which could reduce the need to rebuild models for different sensors or species [133]. It could help address the frozen seafood species- and instrument-specific calibration limitations.

Self-supervised learning has been used to combine data from multiple spectral sources without needing large labeled datasets; its methods learn useful patterns directly from the structure of the unlabeled spectral data itself, which is valuable given how costly and time-consuming labeled freshness or freeze–thaw data collection can be. It could reduce the labeling burden that currently limits many of the deep learning approaches [134].

Transfer learning may help adapt models across different instruments or conditions. Instead of training a new model from scratch for each new instrument, species, or storage condition, transfer learning reuses an existing trained model and fine-tunes it with a smaller new dataset, which can substantially reduce data collection and training time. This has been demonstrated for general food quality models [135], but its use to adapt models specifically across frozen seafood species, instruments, or storage conditions remains largely unexplored.

Federated learning is becoming more common because it allows models to be trained across different facilities without sharing raw data. Instead of sending sensor data to a central location, each facility trains a local copy of the model and only shares the updated model parameters, which addresses data privacy without requiring processors to expose proprietary quality or production data [136]. It could allow different processors, species, or regions to jointly improve model performance without pooling raw data.

Edge AI allows models to run directly on local devices quickly, which is useful for production lines, rather than sending data to a remote server for processing. The model runs directly on the sensor or nearby hardware, which avoids network delays and keeps sensitive data on site, making it well suited to the in-line monitoring gap [133]. It directly addresses the online monitoring limitations for several sensing technologies in this review.

Digital twins are also being developed as tools that combine sensor data with models to predict shelf-life and quality changes during the cold chain; they continuously update a virtual model of the product using real-time sensor data, which allows shelf-life or quality predictions to adjust dynamically as actual storage conditions change, rather than relying on fixed shelf-life estimates set at the start of storage [137]. They could integrate the sensing technologies discussed throughout this review into a single predictive system for the cold chain. As these methods develop further, combining them with sensing technologies could be a useful direction for frozen seafood quality monitoring in the future.

The main artificial intelligence and chemometric approaches used for frozen seafood quality prediction are summarized in Table 2. Classical chemometric methods, including PCA, PLSR, LDA, and SVM, are commonly used for spectral preprocessing, dimensionality reduction, classification, and regression. Machine learning models such as random forest, support vector regression, and gradient-boosting methods improve the prediction of freshness, oxidation, storage time, and freeze–thaw history. Deep learning models, including CNN-based and spectral–spatial approaches, further improve the analysis of hyperspectral and imaging data. Table 2 also summarizes recent advances in explainable AI and multi-sensor data fusion, reflecting the growing emphasis on improving model interpretability, reliability, and practical application in frozen seafood quality monitoring.

## 5. Applications in Frozen Seafood Quality Monitoring

Frozen seafood quality changes in many ways during storage. Freshness loss, volatile formation, texture deterioration, moisture redistribution, and biochemical changes occur at the same time and influence one another [7]. Because of this, practical quality monitoring systems need to evaluate several quality attributes quickly and, where possible, without damaging the product. The following sections summarize the main applications of sensing technologies and artificial intelligence in frozen seafood quality monitoring, focusing on different quality targets, the sensing methods used, the AI approaches applied, and their reported performance.

### 5.1. Freshness and Spoilage Assessment

Freshness and spoilage assessment is one of the most important applications of sensing technologies and artificial intelligence (AI) in frozen seafood. Although freezing slows microbial growth and biochemical deterioration, quality continues to decline during frozen storage and becomes more noticeable after thawing or under temperature-abuse conditions. ATP degradation, protein and lipid oxidation, volatile accumulation, moisture loss, and structural changes all contribute to freshness loss and are commonly evaluated using quality indicators such as K value, total volatile basic nitrogen (TVB-N), thiobarbituric acid reactive substances (TBARS), carbonyl content, Ca^2+^-ATPase activity, color, odor, and texture [7]. Because these quality changes occur together, there is increasing interest in sensing platforms that can evaluate several freshness attributes at the same time rather than relying on a single indicator.

Recent studies show that optical sensing technologies, electronic sensors, and AI algorithms can provide rapid and reliable freshness assessment while overcoming many of the limitations of conventional laboratory methods. Spectroscopic techniques detect biochemical changes related to protein degradation and lipid oxidation, imaging methods capture visible quality changes, while electronic noses and electronic tongues evaluate volatile compounds and taste-related changes that develop during storage. When combined with machine learning or deep learning models, these sensing technologies provide a more complete picture of seafood quality than conventional destructive analyses. Image-based AI methods have also shown good potential for rapid freshness grading after thawing. For example, Genç et al. [127] used machine learning and deep learning models to classify the freshness of frozen–thawed shrimp during refrigerated storage. Their study showed that visual features, including surface color and melanosis-related changes, can support rapid and non-destructive freshness classification in frozen–thawed seafood. Similarly, Wu et al. [143] combined odor imaging with hyperspectral imaging to monitor freshness changes in large yellow croaker. Although this work focused mainly on freshness rather than frozen storage, it demonstrates how different sensing technologies can be combined to improve seafood quality assessment.

These studies show that freshness assessment is gradually moving from single-indicator measurements to integrated sensing systems supported by AI. By combining spectral, visual, odor, and taste information, these approaches provide a more complete evaluation of quality deterioration than conventional methods based on individual chemical indicators. However, further validation under commercial frozen storage conditions, repeated freeze–thaw cycles, and temperature-abuse scenarios is still needed before these technologies can be widely adopted throughout the seafood industry.

### 5.2. Protein, Texture, and Water-Holding Capacity Evaluation

Protein stability, texture, and water-holding capacity (WHC) are closely linked and largely determine the sensory quality and commercial value of frozen seafood. During frozen storage and repeated freeze–thaw (F–T) cycles, ice crystal formation and recrystallization disrupt muscle structure, promote protein denaturation and aggregation, weaken protein–water interactions, and eventually reduce WHC and textural integrity [28,33]. Since these changes occur at the same time, effective evaluation requires sensing technologies that can monitor both protein structural changes and water distribution within muscle tissue. Among the available sensing methods, Raman spectroscopy is especially useful for monitoring protein structural changes because it directly measures molecular vibrations associated with protein secondary structures. Changes in the amide I and amide III regions reflect alterations in α-helix, β-sheet, and random coil structures that occur during protein denaturation in frozen storage [74,107,144]. These structural changes are closely related to reduced protein solubility, lower gel-forming ability, and gradual texture deterioration, making Raman spectroscopy a valuable tool for evaluating protein functionality in frozen seafood [145].

LF-NMR complements Raman spectroscopy by providing direct information on water mobility and protein–water interactions without damaging the sample [146]. Progressive shifts in the T_21_ relaxation peak reflect the movement of immobilized water to the free-water fraction as protein denaturation weakens the myofibrillar network, providing a reliable indication of declining WHC and increasing drip loss during thawing [81,83]. The close relationship between LF-NMR relaxation behavior and moisture migration has been reported in different seafood species, supporting its use as a rapid, non-destructive indicator of structural deterioration.

More recently, sensing technologies have been combined with AI to improve the evaluation of protein- and texture-related quality attributes. The study by Yan et al. [79] showed that hyperspectral imaging combined with a cross-modal deep learning framework accurately identified the freeze–thaw history of large yellow croaker using spectral information collected from fish eyes. Because repeated freeze–thaw cycles accelerate protein denaturation, moisture migration, and structural damage over time, identifying freeze–thaw history also provides useful information about cumulative quality deterioration. Attention-based deep learning architectures further demonstrate the ability of AI to detect subtle structural changes that are difficult to identify using conventional analytical methods. No single sensing technique can fully capture changes in protein integrity, water distribution, and texture. Combining spectroscopic methods, imaging, and AI provides a broader assessment of these quality attributes than any individual sensing technique. Future studies should focus on integrating complementary sensing technologies with explainable AI models that can directly predict functional quality attributes, including water-holding capacity, texture stability, and protein functionality, under practical industrial storage conditions.

### 5.3. Lipid Oxidation and Flavor-Related Quality Monitoring

Lipid oxidation is one of the main causes of quality deterioration in frozen seafood, particularly in fatty fish species. During frozen storage, the oxidation of unsaturated fatty acids produces hydroperoxides, aldehydes, ketones, alcohols, and other volatile compounds that contribute to rancid flavors, undesirable odors, color deterioration, and nutritional losses [147]. Since these chemical changes develop gradually during storage, rapid and non-destructive monitoring of lipid oxidation is essential for quality control and shelf-life management. Spectroscopic techniques combined with artificial intelligence offer an effective way to predict lipid oxidation without relying solely on conventional laboratory assays. Rather than measuring oxidation products directly through destructive methods such as TBARS or peroxide value, spectroscopic techniques detect molecular changes that accompany oxidative deterioration. Guo et al. [53] showed that combining near-infrared (NIR) and Raman spectroscopy with a convolutional neural network (CNN) significantly improved the prediction of TBARS values in frozen salmon stored under both constant and fluctuating temperature conditions. The improved performance was due to the complementary strengths of the two sensing techniques. NIR spectroscopy was more sensitive to changes associated with moisture redistribution and lipid functional groups, whereas Raman spectroscopy better detected structural changes in unsaturated fatty acids and the formation of oxidation products. Combining both techniques therefore provided a more reliable assessment of lipid oxidation than either method alone.

Flavor deterioration extends beyond lipid oxidation. Protein degradation, microbial metabolism after thawing, and interactions among oxidation products also contribute to the complex volatile profile responsible for seafood flavor. Electronic nose (E-nose) systems have become valuable tools for rapidly characterizing volatile organic compound (VOC) patterns generated during frozen storage and thawing [51,87]. However, E-nose responses are pattern-based and cannot identify individual off-flavor compounds. For this reason, flavor assessment should go beyond general proxy indicators such as TVB-N, which reflects overall freshness but cannot fully describe the complexity of flavor deterioration [148]. Recent studies combining GC–MS profiling with lipidomics have shown how specific volatile compounds are associated with lipid oxidation and sensory quality in frozen fish products, including frozen grass carp subjected to freeze–thaw cycles [149]. These integrated approaches improve our understanding of flavor deterioration by linking specific chemical reactions with measurable changes in aroma and consumer perception, bringing flavor prediction closer to practical application.

Research in this area is gradually moving beyond predicting individual quality indicators such as TBARS or TVB-N toward a broader evaluation of flavor deterioration using integrated sensing platforms. Future work will likely combine spectroscopic techniques, volatile profiling, and intelligent data analysis to develop more reliable prediction models that directly relate oxidative chemistry to sensory quality under commercial frozen storage conditions.

### 5.4. Shelf-Life Prediction and Quality Grading

Shelf-life prediction and quality grading are among the most valuable applications of sensing technologies and artificial intelligence (AI) in the frozen seafood industry. Knowing how much shelf life remains helps producers, distributors, and retailers make better decisions during storage and transportation, reducing unnecessary waste while ensuring products reaching consumers meet quality and safety requirements. Unlike conventional approaches that rely on fixed expiration dates, AI-based models estimate product quality dynamically by considering its actual condition throughout the cold chain [23,110].

Recent work has shown that combining spectroscopy with machine learning algorithms can accurately estimate storage time and product quality without destructive testing. For instance, the study by Li et al. [73] combined near-infrared (NIR) spectroscopy with an explainable machine learning model to predict the storage time of frozen Antarctic krill over 12 months of frozen storage. In addition to achieving good prediction accuracy, the study identified the wavelengths that contributed most to the model predictions through SHAP analysis. This not only improved model performance but also provided a clearer understanding of how changes in moisture and protein structure influenced quality during storage. AI has also been integrated with smart packaging technologies. Time–temperature indicators, freshness labels, and intelligent packaging sensors continuously record storage conditions and provide additional information that improves shelf-life prediction [91,93,104,110,150]. When combined with spectroscopic sensing, these technologies make it possible to monitor product quality continuously instead of relying only on predetermined storage periods. This is particularly valuable for frozen seafood because temperature fluctuations and repeated freeze–thaw cycles can accelerate quality deterioration even when products remain within their labeled shelf life.

Despite these advances, several challenges still limit practical application. Most published models have been developed using samples from a single seafood species under carefully controlled laboratory conditions. Their performance under commercial storage and transportation conditions, where temperature, packaging, and product characteristics vary considerably, has received much less attention. In addition, differences among seafood species make it difficult to develop prediction models that perform consistently across a wide range of products. Future studies should therefore validate these models using larger and more diverse datasets collected under real cold-chain conditions while improving their robustness and wider applicability. Sensing technologies combined with AI continue to improve shelf-life prediction and quality grading in frozen seafood. Continued progress in sensor development, explainable AI, and smart packaging is expected to support the transition from traditional date-based shelf-life estimation to real-time quality monitoring throughout the cold chain.

## 6. Challenges and Future Directions

### 6.1. Species- and Product-Specific Quality Deterioration

Different seafood products do not deteriorate in the same way during frozen storage, making quality assessment particularly challenging. Fish, shrimp, shellfish, whole fish, filets, minced products, and surimi differ in lipid content, muscle structure, water distribution, and enzyme activity. As a result, the main quality concern varies from one product to another.

Fatty fish, such as salmon and Atlantic mackerel, are especially susceptible to lipid oxidation during low-temperature or frozen storage because of their high content of unsaturated lipids. Oxidation reduces sensory quality by causing rancidity, off-flavors, color changes, and overall quality deterioration [52,151,152].

Variation can also occur within the same species. For example, Li et al. [153] examined different muscle regions of bighead carp during 180 days of frozen storage and found that quality changes were associated with differences in lipid and protein oxidation among muscle regions. This suggests that a sensing model developed from a single sampling site may not accurately represent the quality of the whole product.

Shrimp provides a similar example. In Pacific white shrimp, repeated freeze–thaw cycles accelerate moisture migration, protein degradation, microstructural damage, and overall quality deterioration. These changes become more pronounced after several freeze–thaw cycles, even when the product still appears acceptable after refreezing [154].

Product form also influences quality assessment. Whole fish, gutted fish, filets, minced fish, and surimi products each have different quality characteristics. Whole chilled or frozen fish generally retain quality longer than gutted or fileted products, although the final outcome depends on species, handling practices, temperature, and storage conditions [155]. In surimi, quality is closely related to gel strength, protein stability, whiteness, and water-holding capacity, while freeze-induced deterioration mainly involves ice crystal formation, protein denaturation, lipid oxidation, and weakening of gel properties [58].

These examples show that a single general model is unlikely to perform well for all frozen seafood products. Future work should focus on developing species- and product-specific models validated with samples from different batches, production origins, product forms, storage temperatures, and freeze–thaw histories. Such models would improve the reliability of sensing and AI-based prediction under practical cold-chain conditions.

### 6.2. Sensor Stability in Cold-Chain Monitoring

Sensor stability remains a major challenge in frozen seafood quality monitoring. Many sensors perform well under laboratory conditions, but real cold-chain environments are far more demanding. During storage and transport, frozen seafood is exposed to low temperatures, high humidity, condensation, surface frost, packaging interference, vibration, and temperature fluctuations. These factors can affect sensor signals and reduce model accuracy.

Electronic noses, electronic tongues, biosensors, flexible sensors, and smart packaging have all been explored for monitoring the freshness of aquatic products during cold-chain logistics. However, their performance can be affected by environmental conditions, signal drift, cross-sensitivity, long-term use, calibration requirements, and limited validation under real operating conditions [11,107,156].

High accuracy under laboratory conditions does not necessarily mean that a sensor will perform equally well in seafood processing plants, transport vehicles, warehouses, or retail freezers. Sensor performance therefore needs to be evaluated under realistic cold-chain conditions rather than only in controlled laboratory environments.

Future work should focus on improving humidity compensation, drift correction, anti-condensation design, low-temperature-resistant sensing materials, and automatic calibration. For frozen seafood, sensor stability should also be assessed together with storage time, packaging type, temperature history, and freeze–thaw exposure. This would improve the reliability of sensing systems for practical industrial applications.

### 6.3. Difficulty Monitoring Coupled Quality Changes

Monitoring frozen seafood quality is challenging because several changes often occur at the same time. Ice crystal growth, water migration, protein oxidation, lipid oxidation, texture loss, color change, and flavor deterioration are closely interconnected. For example, a study on frozen tilapia filets showed that ice crystal formation and protein oxidative denaturation acted together to reduce texture and color stability, while water shifted from bound or immobilized water to the free-water fraction during storage [26]. This interaction is also observed in shrimp and shellfish. In frozen Penaeus monodon, water migration was associated with changes in protein quality indicators and volatile compounds, showing that water loss, protein deterioration, and flavor changes are closely linked rather than occurring independently [51]. Likewise, a study on scallops found that freeze–thaw cycles caused protein oxidation and degradation, leading to muscle fiber breakage and reduced texture quality [29].

These findings highlight why frozen seafood quality cannot be reliably evaluated using a single sensor or quality indicator. Individual indicators, such as TVB-N, TBARS, drip loss, color, or texture, describe only part of the deterioration process. A more reliable monitoring strategy requires combining multiple quality indicators with different sensing methods to capture these interconnected changes. For AI models, the goal should not only be to predict a single quality parameter but also to explain how water migration, oxidation, microstructural damage, and sensory quality change together during frozen storage.

### 6.4. Detecting Thawing, Refreezing, and Temperature Fluctuations

Detecting thawing, refreezing, and temperature fluctuations remains a major challenge in frozen seafood quality monitoring. In real cold-chain systems, seafood may partially thaw during transport, retail display, or household storage before being refrozen. This can damage muscle structure, increase water loss, accelerate protein denaturation, and reduce texture quality. Once the product is refrozen, however, these changes are often difficult to identify by visual inspection, creating challenges for both quality control and fraud detection.

Research has shown that this is a common practical problem. Liu et al. [84] investigated prepackaged Penaeus vannamei subjected to repeated freeze–thaw cycles and found that temperature fluctuations reduced water-holding capacity, promoted protein denaturation, altered texture, and affected freshness indicators. In fish, portable NIR devices have been used to distinguish fresh and frozen–thawed mackerel, including samples exposed to one or two freeze–thaw cycles, with classification rates above 90% [66]. Likewise, NMR combined with multivariate analysis has been used to differentiate fresh and frozen–thawed fish across different species, although additional samples are still needed to improve model robustness [67].

These findings show that future monitoring systems should do more than estimate storage time; they should also identify hidden temperature-abuse history. Validation under realistic cold-chain conditions should include partial thawing, repeated freeze–thaw cycles, different packaging types, and fluctuating storage temperatures. Combining temperature loggers, smart indicators, spectroscopic techniques, electronic nose systems, and AI models could provide a more reliable approach for identifying thawing and refreezing events in frozen seafood.

### 6.5. Limited Transferability of Prediction Models

Limited model transferability remains a major challenge in AI-based frozen seafood quality assessment. Many models achieve high accuracy within a single study, but they are often trained under fixed conditions, such as one species, one storage temperature, one instrument, one imaging background, or one sample batch. For example, Genç et al. [127] reported high classification accuracy for frozen–thawed shrimp freshness, but the samples were collected under controlled refrigerated storage using a fixed imaging setup. Although these results demonstrate good model performance, they do not necessarily show that the model will perform equally well with other shrimp batches, seafood species, cameras, storage temperatures, or industrial environments. A similar limitation has been reported for spectroscopic methods. Giró-Candanedo et al. [66] used low-cost portable NIR devices to distinguish fresh and frozen–thawed mackerel with good classification accuracy. However, the study also showed that models developed from a single batch can be more accurate but less robust because the fish originated from one season and shared similar characteristics. Misclassification may therefore be related to differences among fish or batches collected in different seasons. This is particularly important for frozen seafood, as product composition can vary with season, origin, size, fat content, processing method, and freezing history.

Another factor affecting transferability is the sensitivity of spectral and imaging signals to external conditions. In food quality control, calibration transfer is commonly used to reduce the loss of model performance caused by changes in instruments, temperature, humidity, orientation, and measurement conditions. Studies on visible/NIR spectroscopy have also shown that calibration models may lose predictive ability when different instruments or external conditions are introduced, limiting their application on a larger scale [111].

Model evaluation should therefore extend beyond internal accuracy and cross-validation. Prediction models need to be tested using independent samples representing different batches, seasons, origins, product forms, instruments, and storage conditions. Transfer learning, calibration transfer, and regular model updating may improve robustness, but these approaches still require external validation. For frozen seafood, a reliable prediction model should perform consistently not only under laboratory conditions but also in factories, warehouses, transport vehicles, and retail freezers.

### 6.6. Practical Difficulties in Multi-Sensor Fusion

Multi-sensor fusion has attracted considerable interest because frozen seafood quality is influenced by multiple changes occurring simultaneously, including water loss, oxidation, texture deterioration, color changes, and the formation of volatile compounds. By integrating complementary information from gas sensors, imaging systems, spectroscopic techniques, and three-dimensional (3D) data, multimodal data fusion can provide a more comprehensive assessment of food quality than any single sensing technique alone [131].

Despite these advantages, implementing multi-sensor fusion in practice remains challenging. Different sensors generate data with different dimensions, noise characteristics, acquisition speeds, and preprocessing requirements. If these datasets are not properly aligned, normalized, and selected, combining them may increase model complexity without delivering meaningful improvements in prediction accuracy [157]. For example, Wang et al. [158] integrated hyperspectral imaging with electronic nose data to predict the moisture content of Penaeus vannamei. Although the fusion strategy improved overall prediction performance, the authors also reported that the contribution of the electronic nose was limited and that its measurements were sensitive to humidity. These findings highlight that adding more sensors does not necessarily translate into better performance.

Rather than increasing the number of sensing modalities, multi-sensor fusion should be designed around clearly defined quality objectives. The selection of sensors should reflect the specific quality attribute being monitored. For instance, NMR and hyperspectral imaging are particularly valuable for evaluating water migration and texture-related changes, whereas electronic noses and colorimetric sensors are better suited for monitoring volatile compounds and spoilage-related odors. For industrial implementation, fusion models should remain simple, robust, computationally efficient, and easy to operate. Greater emphasis should therefore be placed on sensor selection, data alignment, feature reduction, model interpretability, and validation under realistic frozen seafood cold-chain conditions.

### 6.7. Industrial Cost, Speed, and Usability

Industrial implementation remains one of the final challenges for frozen seafood quality monitoring. A sensing method may perform well under laboratory conditions, but successful industrial adoption also depends on affordability, speed, robustness, and ease of operation. In seafood processing plants, cold-storage warehouses, transportation vehicles, and retail freezers, quality inspection cannot rely on lengthy sample preparation, expensive instrumentation, or highly specialized personnel. Although advanced sensing technologies provide excellent analytical performance, many still face practical limitations. Hyperspectral imaging, for example, offers rich chemical and spatial information, but its high equipment cost, large data volumes, and computationally intensive analysis continue to restrict routine industrial use [77,79,159]. Electronic nose and electronic tongue systems are generally faster, more portable, and easier to deploy, yet their wider adoption is still constrained by sensor drift, the lack of standardized protocols, relatively high costs, and the need for extensive validation under real operating conditions [11,116].

For successful industrial deployment, monitoring systems should be designed with practical application rather than laboratory performance alone in mind. Portable NIR spectrometers, simplified imaging systems, colorimetric labels, time–temperature indicators, and low-cost gas sensors are often more suitable for rapid on-site screening. More sophisticated techniques, such as hyperspectral imaging and low-field NMR, may be better reserved for detailed quality evaluation, model development, or high-value seafood products. Ultimately, the most effective industrial solution is not necessarily the most technologically advanced one, but the one that delivers reliable results rapidly, at a reasonable cost, and in a format that operators can interpret and apply with confidence.

## 7. Conclusions

Frozen seafood quality deteriorates through a series of closely interconnected physical, chemical, and structural changes that occur throughout frozen storage and thawing. Ice crystal formation, water migration, protein denaturation and oxidation, lipid oxidation, texture softening, color deterioration, and flavor loss do not occur independently but continuously influence one another. Because these quality changes develop simultaneously, relying on a single quality indicator rarely provides a complete assessment of product conditions. This complexity explains why non-destructive sensing technologies, combined with artificial intelligence (AI) and multi-sensor data fusion, have become increasingly important for frozen seafood quality monitoring. Over the past decade, remarkable progress has been made in both sensing technologies and intelligent data analysis. Techniques such as near-infrared (NIR) and Raman spectroscopy, hyperspectral imaging, fluorescence imaging, low-field nuclear magnetic resonance (LF-NMR), electronic nose (E-nose), electronic tongue (E-tongue), biosensors, and colorimetric sensing each provide different but complementary information about frozen seafood quality. At the same time, machine learning and deep learning models have greatly improved the ability to interpret these complex datasets. Deep learning models, particularly convolutional neural networks (CNNs), have demonstrated excellent performance for spectral and imaging analysis, while conventional chemometric and machine learning methods remain valuable because of their interpretability and effectiveness with relatively small datasets. More recently, explainable AI has helped bridge the gap between prediction accuracy and scientific understanding by identifying the spectral and sensor features that influence model decisions. Integrating multiple sensing techniques within a single AI framework has further improved prediction performance by providing a more comprehensive representation of seafood quality than any individual sensing method alone.

The studies reviewed in this paper clearly demonstrate that these integrated approaches can successfully evaluate multiple aspects of frozen seafood quality, including freshness, spoilage, protein denaturation, water-holding capacity, lipid oxidation, flavor deterioration, shelf-life prediction, and product grading. Together, these advances show that intelligent sensing is moving beyond proof-of-concept laboratory research toward practical applications in industrial quality control. Nevertheless, several important challenges remain before widespread commercial adoption can be achieved.

Future research should focus on developing larger and more diverse datasets that include different seafood species, product forms, production batches, geographical origins, and realistic cold-chain conditions. Such datasets are essential for building models that remain accurate when applied outside the laboratory. Greater attention should also be given to explainable AI, physics-informed learning, transfer learning, calibration transfer, and robust external validation to improve model reliability across different instruments and processing environments. At the same time, sensing systems should become more affordable, portable, and easier to operate so that they can be adopted by seafood processors of different sizes rather than remaining confined to research laboratories. Establishing standardized validation protocols and regulatory guidelines will also be critical for building confidence in AI-assisted quality assessment and supporting future industrial implementation. Major research gaps include the lack of standardized datasets and the limited reproducibility of sensing and AI models across seafood species, production batches, instruments, and storage conditions.

Looking ahead, the greatest advances are likely to come not from individual sensing technologies, but from their seamless integration into intelligent digital cold-chain management systems. Combining sensing platforms with edge AI, Internet of Things (IoT) connectivity, cloud computing, and blockchain-based traceability has the potential to provide continuous, real-time, and transparent monitoring throughout the entire seafood supply chain. Such systems could improve shelf-life management, reduce food waste, strengthen food safety through earlier detection of quality deterioration, and enhance consumer confidence through reliable product traceability. Achieving this vision will require close collaboration among researchers, technology developers, seafood producers, regulators, and industry stakeholders. Although important challenges remain, the rapid progress made in sensing technologies and artificial intelligence suggests that intelligent, non-destructive quality monitoring is well positioned to become a key component of future frozen seafood quality management.

## Figures and Tables

**Table 1:** Comprehensive comparison of non-destructive sensing technologies for frozen seafood quality monitoring.

Technology	Quality Target	Accuracy	Speed	Cost	Portability	Ind. Readiness	limitations	Refs
NIR Spectroscopy	Water, lipids, proteins (C–H, O–H, N–H overtones and combination bands)	Medium–High	Very fast	Low	High	High	Indirect measurement, sensitive to surface condition, requires calibration	[66,73]
Raman Spectroscopy	Protein secondary structure (amide I, III), lipid unsaturation (C=C stretching, ~1660 cm^−1^), myoglobin oxidation state (ν_4_ porphyrin, ~1374 cm^−1^)	High	Medium	Medium–High	Medium	Low– Medium	Weak signal (low Raman cross-section), fluorescence interference, higher cost	[53,74,76]
HSI/MSI	Spatial distribution of TVB-N, K-value, color, texture, moisture migration, freeze–thaw history	High	Slow	High	Low– Medium	Medium	Data-intensive (large file sizes), slow acquisition, expensive instrumentation, complex processing	[106]
Fluorescence Spectroscopy and Imaging	Aromatic amino acids (tryptophan, tyrosine), NADH, porphyrins, Schiff bases (lipid oxidation products); spatial distribution of oxidation	Medium–High	Medium	Medium–High	Low– Medium	Low	Photobleaching, temperature-sensitive, complex data, specialized instrumentation for imaging	[16,68]
LF-NMR	Water distribution and mobility: T_2_b (bound water, ~0.1–10 ms), T_21_ (immobilized water, ~10–100 ms), T_22_ (free water, >100 ms)	High	Medium	High	Low	Low	Expensive instrumentation, large magnet, sensitive to temperature and magnetic field homogeneity	[81,83]
E-nose	Volatile organic compounds (VOCs): aldehydes (hexanal, propanal), ketones (2,3-butanedione), alcohols (1-octen-3-ol), sulfur compounds (dimethyl sulfide, H_2_S), biogenic amines (trimethylamine, putrescine)	Medium–High	Fast	Medium	High	Medium	Sensor drift over time, humidity/temperature sensitivity, limited individual analyte specificity	[87,107]
E-tongue	Taste-active dissolved compounds: free amino acids (umami), nucleotides (IMP, inosine, hypoxanthine), organic acids, salts, bitter substances	Medium–High	Fast	Medium	High	Medium	Sample extraction (aqueous) often required, sensor fouling over time	[20]
CSAs and Freshness Indicators	Spoilage-related gases: amines (biogenic amines, NH_3_, trimethylamine), sulfur compounds (H_2_S), aldehydes, organic acids, TVB-N	Medium	Fast– Continuous	Low	High	Medium–High	Semi-quantitative, limited shelf life of dye materials, affected by light/humidity, calibration required for quantitative use	[90,91,93]
Biosensors	Specific freshness-related biomarkers: hypoxanthine, histamine, biogenic amines (cadaverine, putrescine)	High	Fast– Medium	Medium	Medium–High	Low– Medium	Stability during cold storage, matrix interference in complex seafood, single-analyte typically	[107]
Ultrasound	Internal structural changes: ice crystal-induced disruption, protein aggregation, tissue density/elasticity	Medium	Fast	Medium	Medium	Low– Medium	Current evidence stronger for process improvement (ultrasound-assisted freezing) than routine monitoring; limited validation	[102,108,109]
TTIs/RFID smart tags	TTIs: cumulative thermal history (time–temperature integral); RFID: temperature, humidity, location	Medium (indirect)	Continuous	Low (TTI)/High (RFID)	Built-in	High	TTIs: require validated kinetic model per product, calibration; RFID: higher cost (active tags), measures environment not direct product quality, power requirements	[110]

**Table 2:** Comprehensive summary of artificial intelligence and chemometric approaches for frozen seafood quality prediction.

AI/Chemometric Method	Category	Typical Application	Data Type Compatibility	Key Strengths	Limitations	References
Principal Component Analysis (PCA)	Unsupervised chemometric	Exploratory analysis, outlier detection, pattern visualization, variance structure evaluation	NIR, Raman, HSI, E-nose, E-tongue	Reduces dimensionality, visualizes clustering, no labeled data required, identifies spectral regions associated with quality changes	No prediction capability, linear only, does not encode quality attributes	[138]
Partial Least Squares Regression (PLS-R)	Supervised chemometric (regression)	Quantitative prediction of TVB-N, TBARS, K-value, microbial counts, storage time	NIR, Raman, HSI, LF-NMR	Handles multicollinearity, interpretable loadings, well-established, bilinear, industry standard	Linear assumption, requires reference measurements for calibration, sensitive to outliers	[73,135]
Partial Least Squares Discriminant Analysis (PLS-DA)	Supervised chemometric (classification)	Classification: fresh vs. frozen–thawed, freshness grade, spoilage detection, species authentication	NIR, Raman, HSI	Extension of PLS for classification, probabilistic outputs, intuitive decision boundary	Linear decision boundary, sensitive to class imbalance, requires balanced datasets	[66]
Support Vector Machines/Support Vector Regression (SVM/SVR)	Classical machine learning (classification/regression)	Freshness grade classification, spoilage detection, freeze–thaw cycle identification, quality indicator prediction	Spectra, extracted features, sensor arrays, HSI features	Effective for high-dimensional spectral/sensor data, handles nonlinear relationships through kernel functions, performs well with limited samples	Requires parameter tuning, less interpretable than PLS models, computationally intensive for large datasets	[20]
k-Nearest Neighbors (kNNs)	Classical machine learning (classification)	Similarity-based classification of quality grades, freshness categorization	Spectral features, sensor response patterns, extracted image features	Simple, non-parametric, intuitive, no training phase, easily updated with new data	Sensitive to irrelevant features, distance metric selection critical, slow for large datasets, affected by class imbalance	[65]
Random Forest (RF)	Ensemble machine learning (classification/regression)	Feature importance ranking, quality classification, shelf-life prediction, spoilage detection	Sensor arrays, spectral features, extracted variables, multi-sensor data	Handles nonlinearity, robust to overfitting, provides feature importance, no scaling needed, handles missing data	Less interpretable than linear models, large ensembles can be slow, memory-intensive	[127]
Artificial Neural Networks (ANNs)	Classical neural network (regression/classification)	Quantitative prediction of quality indices, pattern recognition, nonlinear modeling	Tabular features, sensor fusion, preprocessed spectra, extracted features	Universal approximator, captures complex nonlinear relationships, flexible architecture	Prone to overfitting, requires large data, black box, architecture selection critical, slow training	[65]
Convolutional Neural Networks (CNNs)	Deep learning (image/spectral analysis)	HSI data cube analysis, RGB image classification, spectral feature extraction, freshness grading, defect detection	HSI cubes (3D), 2D images, 1D spectra, RGB images	Automatic hierarchical feature extraction, captures spatial–spectral patterns, end-to-end learning, no manual feature engineering	Computationally intensive, requires large labeled datasets, black box, GPU recommended, long training time	[127,139]
Domain Adaptation	Machine learning strategy	Model generalization across instruments or facilities (calibration transfer), cross-species generalization	Spectra from multiple instruments, data from different facilities	Aligns feature distributions across domains, enables deployment without full recalibration, reduces annotation burden	Requires some target-domain data, algorithmic complexity, domain shift assumptions	[129,140]
Explainable AI (XAI)–SHAP	Post hoc interpretation (model-agnostic)	Identifying which spectral bands, image regions, or sensor channels drive predictions; feature importance ranking	Any model output (model-agnostic), works with black-box models	Game-theoretic foundation, consistent local explanations, identifies global and local feature importance, unified framework	Computational overhead (especially for large models), post hoc approximations, sampling approximations	[73,127]
Grad-CAM	CNN-specific interpretation	Visual saliency maps showing which image regions or spectral bands influence CNN predictions	CNN outputs (gradient-based), HSI images, RGB images	Visual heatmaps overlaid on input images, intuitive for HSI/RGB, class-discriminative	Only works for gradient-based models (CNNs), not model-agnostic, requires backpropagation	[127,141]
Physics-Informed AI	Hybrid modeling (emerging)	Incorporating known physicochemical mechanisms (kinetics, spectroscopic signatures, oxidation pathways) into model architecture	Any data type, with mechanistic priors, spectral data with known absorption bands	Simultaneously accurate and interpretable, physically grounded, regulatory friendly, improved generalization	Requires mechanistic knowledge, more complex model design, emerging methodology	[142]
Multi-Sensor Data Fusion—Low-Level	Data fusion strategy	Combining raw or preprocessed signals from multiple sensors before modeling; maximum information preservation	Multiple sensor modalities (e.g., NIR + Raman, E-nose + E-tongue, HSI + colorimeter)	Preserves all original information, simple concatenation, no information loss, end-to-end learning possible	High dimensionality, redundant features, alignment/synchronization required, potential overfitting	[19,53,138]
Multi-Sensor Data Fusion—Mid-Level	Data fusion strategy	Extracting features from each modality separately before integration; dimensionality reduction	Multiple sensor modalities (e.g., NIR + Raman, E-nose + E-tongue + colorimeter)	Reduces dimensionality, removes redundancy, flexible, interpretable features	Feature extraction design critical, potential information loss, two-stage process	[20,138]
Multi-Sensor Data Fusion—High-Level	Data fusion strategy (ensemble)	Combining independent outputs (decisions) of modality-specific models; voting or weighting schemes	Multiple sensor modalities (any combination)	Robust to modality failure, simple to implement, voting/weighting schemes, modular	No interaction between modalities during feature learning, potential information loss from each modality	[138]
Competitive Adaptive Reweighted Sampling (CARS)	Feature selection (chemometric)	Selecting informative wavelengths/variables from high-dimensional spectral data; removing uninformative variables	NIR, Raman, HSI spectra, high-dimensional spectral data	Reduces input dimensionality, improves model interpretability, identifies key spectral bands, automated selection	Wavelength selection can be unstable, requires calibration, random sampling component	[65]
Successive Projections Algorithm (SPA)	Feature selection (chemometric)	Selecting wavelengths with minimal collinearity for MLR/PLS; forward selection with projection	Spectra, high-dimensional collinear data	Eliminates redundant variables, simple forward selection, good for linear models, deterministic	May miss nonlinear interactions, greedy algorithm (may not find global optimum), linearity assumption	[65]

## Data Availability

No new data were created or analyzed in this study. Data sharing is not applicable to this article.
